# *Mycobacterium tuberculosis* Proteins Involved in Mycolic Acid Synthesis and Transport Localize Dynamically to the Old Growing Pole and Septum

**DOI:** 10.1371/journal.pone.0097148

**Published:** 2014-05-09

**Authors:** Clément Carel, Kanjana Nukdee, Sylvain Cantaloube, Mélanie Bonne, Cheikh T. Diagne, Françoise Laval, Mamadou Daffé, Didier Zerbib

**Affiliations:** 1 Centre National de la Recherche Scientifique, Institut de Pharmacologie et de Biologie Structurale, Toulouse, France; 2 Université de Toulouse, Université Paul Sabatier, Toulouse, France; Université de Montpellier 2, France

## Abstract

Understanding the mechanism that controls space-time coordination of elongation and division of *Mycobacterium tuberculosis* (*Mtb*), the causative agent of tuberculosis (TB), is critical for fighting the tubercle bacillus. Most of the numerous enzymes involved in the synthesis of Mycolic acid - Arabinogalactan-Peptidoglycan complex (MAPc) in the cell wall are essential *in vivo*. Using a dynamic approach, we localized *Mtb* enzymes belonging to the fatty acid synthase-II (FAS-II) complexes and involved in mycolic acid (MA) biosynthesis in a mycobacterial model of *Mtb*: *M. smegmatis*. Results also showed that the MA transporter MmpL3 was present in the mycobacterial envelope and was specifically and dynamically accumulated at the poles and septa during bacterial growth. This localization was due to its C-terminal domain. Moreover, the FAS-II enzymes were co-localized at the poles and septum with Wag31, the protein responsible for the polar localization of mycobacterial peptidoglycan biosynthesis. The dynamic localization of FAS-II and of the MA transporter with Wag31, at the old-growing poles and at the septum suggests that the main components of the mycomembrane may potentially be synthesized at these precise foci. This finding highlights a major difference between mycobacteria and other rod-shaped bacteria studied to date. Based on the already known polar activities of envelope biosynthesis in mycobacteria, we propose the existence of complex polar machinery devoted to the biogenesis of the entire envelope. As a result, the mycobacterial pole would represent the Achilles' heel of the bacillus at all its growing stages.

## Introduction

The mycobacterial cell envelope is of an exceptional complexity. One of the major challenges for *Mycobacterium tuberculosis* (*Mtb*) is probably achieving the synthesis of this thick multilayer barrier [1], [2]. The *Mtb* envelope is partly responsible for its innate resistance to antibiotics and plays a major role in both its virulence and persistence. Tuberculosis (TB) reappeared in the 1990s and remains one of the main causes of lethality worldwide. The principal challenge is to fight the increasing number of multi-drug resistant (MDR) *Mtb* clinical isolates. The alternate periods of growth and dormancy of *Mtb* during infection [3] render the discovery of new effective antibiotics difficult. *Mtb* division, as well as the regulation of its cell cycle and the maintenance of cell wall homeostasis, are nonetheless weak points of the bacillus. Understanding the mechanisms that guarantee spatial and temporal coordination of *Mtb* envelope biogenesis during the cell cycle is a crucial step toward deciphering the role of this major player of *Mtb* pathogenesis [4], [5].

In *Mtb*, two Fatty-Acid-Synthase systems (FAS), FAS-I [6] and FAS-II [7], are devoted to the synthesis of common fatty acids and of specific, long-chain, α-acylated, β-hydroxylated fatty acids, namely mycolic acids (MA) [8], [9]. Mycolic acids constitute the major lipid component of the envelope and form the external mycomembrane [10], [11]. They are either inserted in the mycomembrane as trehalose dimycolates and monomycolates (TDM and TMM) or linked covalently to the underlying arabinogalactan (AG), itself bound to peptidoglycan (PG) to form the MAPc [12], [13]. Mycolic acids stem from the condensation of a medium chain-length fatty acid (C_24_-C_26_) produced by FAS-I with a long meromycolic chain (up to C_60_) produced by FAS-II (Figure S1) [14], [15]. FAS-II is comprised of specialized and interconnected protein complexes [16], [17], [18] (Figure S2). The MA biosynthetic pathway is assumed to be cytoplasmic, a notion recently reinforced by the discovery of a MA transporter in mycobacteria: the potential trans-membrane protein MmpL3 [19], [20], [21]. We hypothesized that at least part of the FAS-II multi-protein complexes may exist as stable entities and could be involved in the biogenesis of MAPc during bacterial growth.

**Figure 1:**
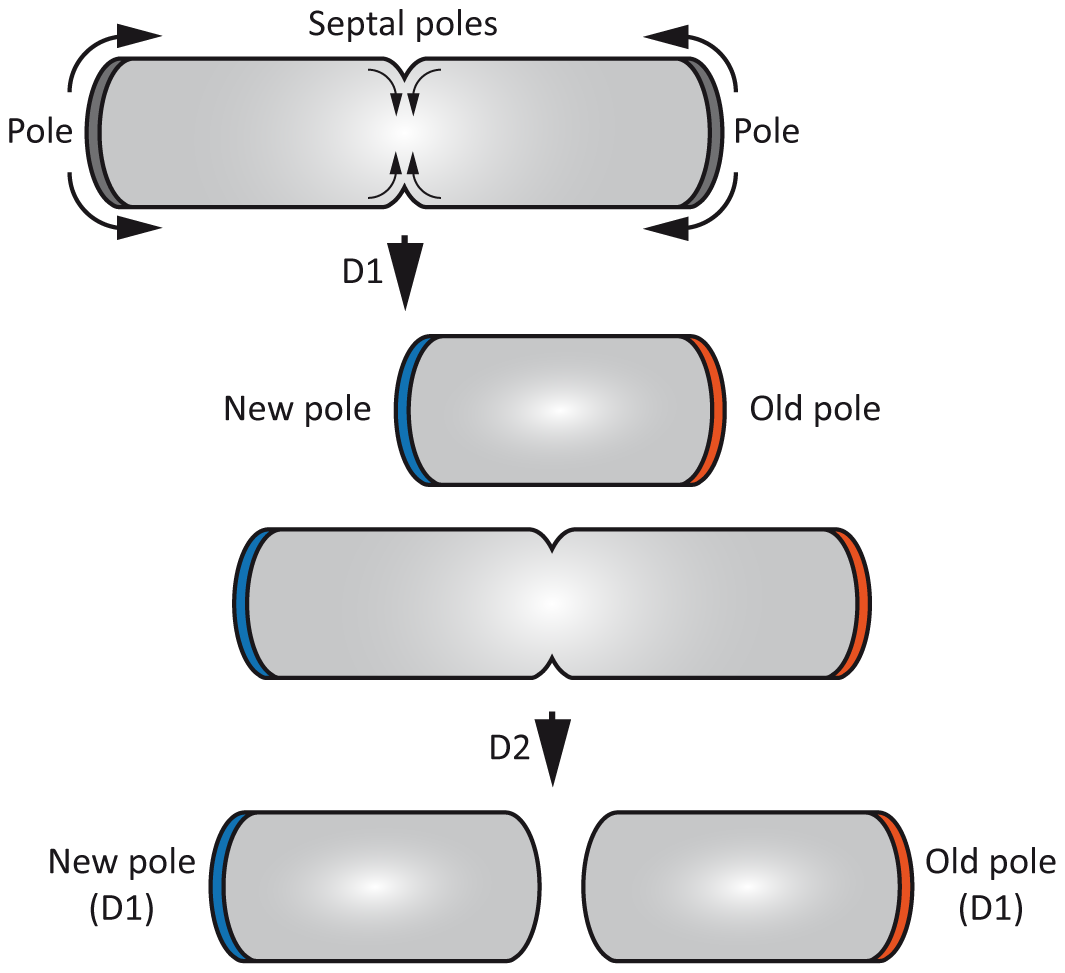
Schematic representation of mycobacterial polar growth and establishment of bacterial poles. Two division cycles (D1 and D2) are presented. The active biosynthesis of lateral peptidoglycan at the poles and biosynthesis of septal peptidoglycan at the future pole (septal poles) are symbolized with curved arrows. The old pole (in red) and the new pole (in blue); are both represented and refer to the first division event (D1).

The cell wall elongation process of *actinobacteria* [4] is different from that of other rod-shaped models such as *Escherichia coli* or *Bacillus subtilis* [22]. In the latters, nascent PG is made all along the side of the bacterium during elongation and at the septum during the division process [23] whereas in the former, the lateral PG derives from polar biosynthesis during elongation [24]. Among mycobacteria, the polar localization of PG synthesis is mainly due to the phospholipid-interacting protein Wag31, the ortholog of the *B. subtilis* DivIVA protein [25], [26], [27]. Wag31, by targeting negatively-curved membranes [28], is responsible for the polar localization and the septal re-localization of several PG biosynthesis proteins [29], [30]. Wag31 is mainly located at the “old pole” (Figure 1), which is the active locus among mycobacteria and other *actinobacteria* where new molecular material of the lateral cell wall is added [31], [32]. The “septal pole” (Figure 1), which represents the future pole and the developing septum, does not participate in lateral PG biosynthesis [33].

We demonstrate herein that reductases from the FAS-II core as well as the MA transporter MmpL3 are located at the active mycobacterial old poles and at the position of the septum prior to division. The condensing enzymes form polar foci but are also able to diffuse in the cytoplasm. FAS-II localization is correlated with the dynamics of cell division, with the localization of Wag31 and with the dynamics of the MA transporter MmpL3. These results describe the first observation of the localization of sophisticated machinery for fatty acid biosynthesis. In addition, they reveal the existence of a possible link between two central and essential processes: bacterial division and whole envelope biogenesis. Mycolic acids may be synthesized at the active poles and septa in order to be transported, probably by MmpL3, to the external mycomembrane.

## Results

### FAS-II complex components are located at one bacterial pole

**Figure 2:**
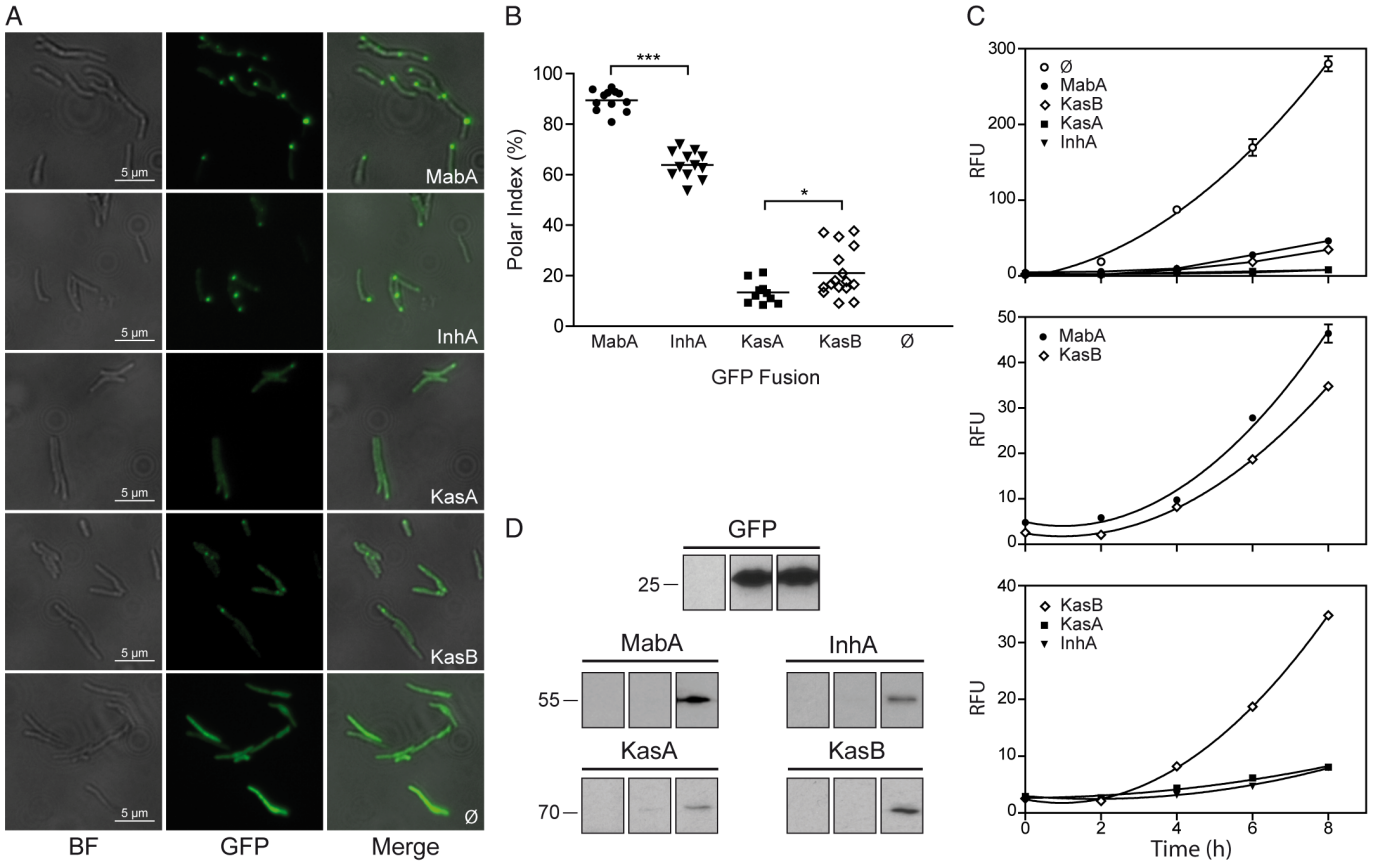
Polar localization of FAS-II proteins. (A) Enlarged images of wide-field microscopy experiments on GFP-fusion expressing bacteria. Bright field images (BF; leftmost column) together with GFP fluorescence images (GFP, middle column) of *Msm* expressing GFP fusions with MabA, InhA, KasA or KasB, or GFP alone (Ø) were acquired after 6 hours of induction. The merged images (right columns) allowed visualizing the polar localization of the fusions. Total optical magnification: 630 X. Scale bar: 5 µm. (B) Scatter dot plot representation of polar indexes of *Msm* strains expressing each GFP fusion after 6 hours of induction. Data were collected from wide-field microscopy images and subsequently expressed and evaluated as described in Materials and Methods. Statistical t-tests were performed to assess the differences between the polar indices of GFP-MabA (black circles) and GFP-InhA (black triangles) and between GFP-KasA (black squares) and GFP-KasB (open diamonds). Each data point corresponds to an individual experiment. Each series of data points for a given fusion represents 500 to1500 bacteria analyzed. The *p* values of indicated unpaired t-tests are symbolized by asterisks (***, *p*<0.0001), (*, *p* = 0.0264). (C) Total fluorescence of *Msm* cultures expressing GFP fusions. Experimental values are represented as means ± SEM. Fluorescence intensities, expressed in RFU (as defined in the text and in the Materials and Methods) of liquid cultures of *Msm* expressing either GFP alone (Ø), GFP-MabA (black circles), GFP-KasB (open diamonds), GFP-KasA (black squares) or GFP-InhA (black triangles) were plotted against the length of induction in hours (h). The curve fit was obtained by using a second order polynomial nonlinear regression (quadratic) calculated with GraphPad Prism 5.0 software. (D) Western Blot analysis of GFP-fusions. Total protein content of *Msm* culture samples expressing either GFP alone or the GFP-fusions with the indicated proteins was analyzed by Western blotting using anti-GFP antibodies. The blots were performed 2 hours (left lanes), 4 hours (middle lanes) or 6 hours (right lanes) after acetamide induction of the same cultures depicted in panel C. The molecular weights of the relevant marker bands are indicated in kDa.

*Mtb* genes encoding the FAS-II reductases MabA and InhA, and the condensing enzymes KasA and KasB, were merged to the C-terminal end of *gfp* and expressed under the control of the P_amiE_ inducible promoter in the non-pathogenic bacterium model *M. smegmatis* mc^2^155 (*Msm*). In a first series of experiments, large field fluorescence microscopy images, hereafter called static images, were acquired six hours after induction. GFP alone emitted a bright and diffuse fluorescence throughout the cytoplasm (Figure 2A). Conversely, the reductase fusions yielded intense foci located mainly at one bacterial pole (Figure 2A). In the strains expressing either GFP-KasB or GFP-KasA, polar foci were also detectable although together with a diffuse fluorescence in the cytoplasm (Figure 2A). To quantify foci formation, a polar index (PI) was calculated and defined as the ratio of the number of bacteria containing at least one polar focus reported to the total number of fluorescent bacteria (N). Over one thousand bacteria expressing each fusion were analyzed on several replicates. Two groups were found (Figure 2B). The first group, e.g. MabA (89.47%±1.20, N = 1100) and InhA (63.86%±1.56, N = 2000), had a high PI while the second group, e.g. KasA (13.34%±1.40, N = 900) and KasB (21.02%±2.40, N = 1700), had a lower PI. Due to the inaccuracy of quantifying and comparing static images from different snapshots, the total amount of fluorescence in live bacterial culture was assessed by fluorometry as a function of induction time. This enabled to verify whether PI was correlated or not with fusion expression levels. Eight hours after induction (Figure 2C), the strain containing GFP alone (PI = 0%) emitted the maximum fluorescence (280 RFU). MabA (PI = 89%) and KasB (PI = 21%) yielded intermediate fluorescence levels (45 RFU and 35 RFU, respectively) whereas KasA (PI = 13%) and InhA (PI = 63%) yielded very low levels (below 10 RFU). The absence of correlation between fluorescence levels and PI values (as compared between Figure 2B and Figure 2C) was further confirmed by Western blotting experiments performed on the same cultures (Figure 2D). Western blotting results were perfectly correlated with fluorometry results (see comparison between Figure 2D and Figure 2C) and thus were not correlated with PI values, indicating that foci formation was not due to protein overexpression but rather due to preferential localization of FAS-II at the pole.

### Dynamics of polar localization of FAS-II proteins

**Figure 3:**
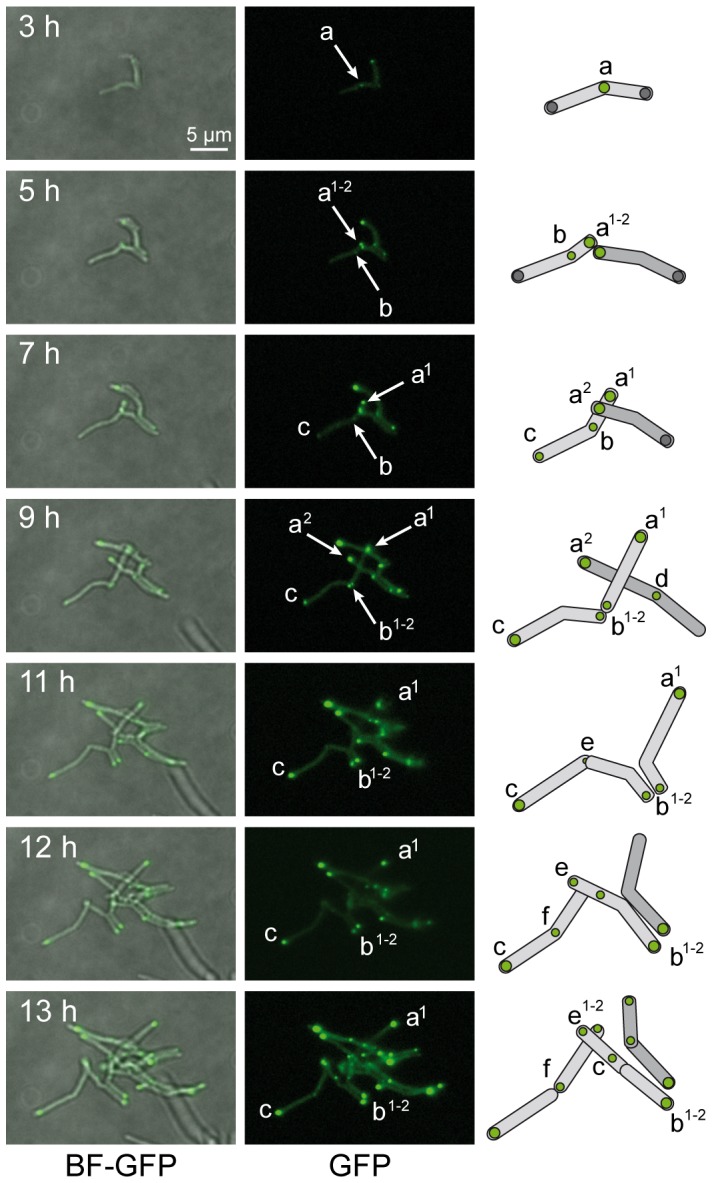
Dynamics of GFP-MabA localization. Images were extracted from the movie presented as movie S2. The time interval after the starting point of the movie is indicated in hour (h). Merge bright field and GFP channel images (BF-GFP, left column) and GFP channel images (GFP, right column) were extracted in order to follow the dynamics of representative GFP-MabA foci. Two bacteria and their daughter cells were schematized (far right). Representative foci (in green) are labeled (a to f) and followed, when possible, in the daughter cells. Superscript numbering represents potential foci splitting. White arrows indicate the localization of the main foci on GFP images. Magnification and scale are identical to those indicated in Figure 2A.

In order to analyze foci formation and localization and avoid potential problems due to protein accumulation, a dynamic analysis was implemented. The goal was to observe and follow the very early stages of foci formation during bacterial division, starting just after the beginning of the induction of GFP-fusion expression with a low amount of inducer (0.02% acetamide). Time-lapse microscopy experiments were designed using a recent and efficient agar pad microscopy technique [34]. In the GFP-producing strain (Movie S1), the signal was bright and diffuse in which no foci were observed. In the GFP-MabA producing strain (Movie S2), at the beginning of induction, the first foci (Figure 3, foci a, 3 h; and b, 5 h), were observed at the septal poles of the mother bacterium. These foci appeared at mid-cell at the position of the next division septum. These types of foci were often apparently split in two after the end of the division process (Figure 3, foci a^1-2^, 5 h; b^1-2^, 9 h). Thereafter, they became established at the new poles of the first pair of daughter bacteria (Figure 3, foci a, b, c, 9 h to 13 h). At the end of induction the images resembled the static images from the first experiments (Figure 2A) where this protein was present at only one pole, leading to conclude that these foci correspond to dynamic protein complexes. The same results were obtained with the InhA fusions (Movie S3), displaying foci which were first visible at the septal poles and thereafter establishing at one preferential pole. Because of the lower level of expression of the KasA fusion (Movie S4), it was more difficult to follow foci dynamics at the beginning of induction although in the micro-colonies, GFP-KasA foci were also first visible at the septal poles and then appeared very rapidly at the poles. Finally, the KasB fusion (Movie S5) was diffuse in the cytoplasm at the early stage of induction and the foci were only visible at one pole at the end of the experiment. There was no typical envelope labeling by FAS-II fusions observed at any time, indicating that the FAS-II protein tested are probably cytoplasmic. We thus hypothesized that these proteins ultimately participate in pole structure in order to ensure elongation of the lateral mycomembrane.

### FAS-II proteins are localized with the old pole marker protein Wag31

**Figure 4:**
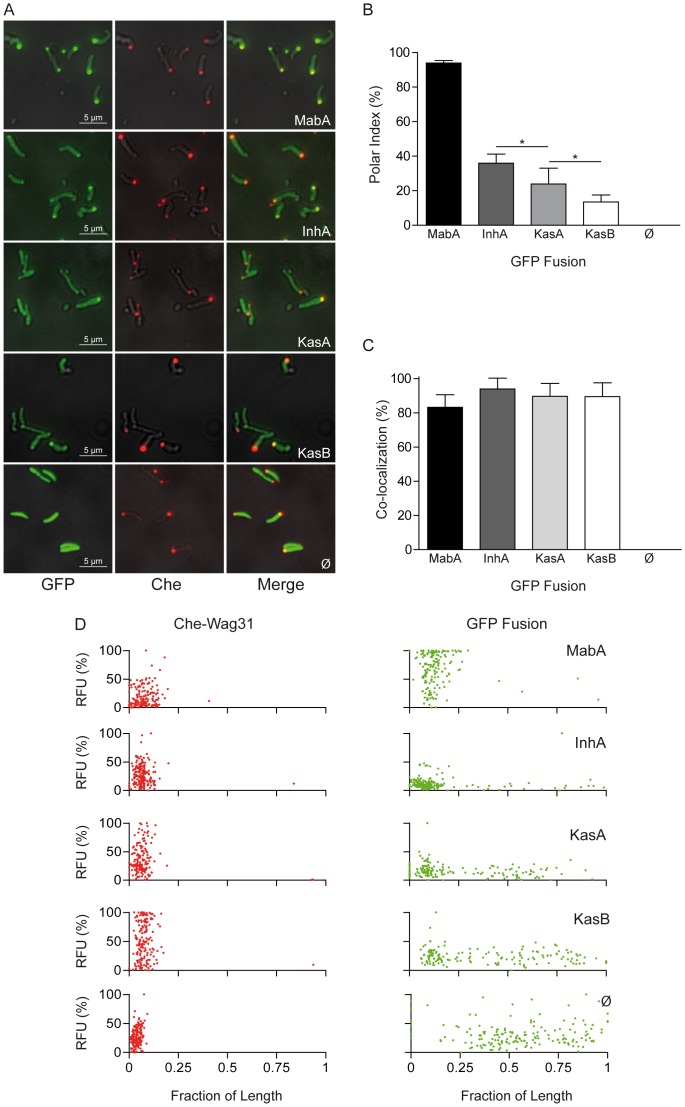
Polar colocalization of FAS-II proteins with Wag31. (A) Enlarged images of wide-field microscopy experiments on GFP-fusion and mCherry-Wag31 fusion-expressing bacteria. GFP fluorescence images (GFP; leftmost column) together with mCherry fluorescence images (Che, middle column) of *Msm::mCherry-wag31* expressing GFP fusions with MabA, InhA, KasA, KasB, or GFP alone (Ø) were acquired after 6 hours of induction. The merged images (right columns) allowed visualizing the polar colocalization of the fusions. Magnification and scale bars are identical to those indicated in Figure? 2A. (B) Bar graph representation of GFP polar indices of *Msm::mCherry-wag31* strains expressing each GFP fusion after 6 hours of induction. Experimental values are represented as mean ± SD. Data were processes as described in Figure? 2B. Statistical *t*-tests were performed to determine the differences between the polar indices of GFP-InhA (dark grey bar) and GFP-KasA (light grey bar) and of GFP-KasA and GFP-KasB (white bar). The *p* values of indicated unpaired t-tests are symbolized by asterisks (*, *p* = 0.0271 for InhA-KasA), (*, *p* = 0.0186 for KasA-KasB). (C) Bar graph representation of GFP-mCherry colocalization indices of *Msm::mCherry-wag31* strains expressing each GFP fusion after 6 hours of induction. Experimental values are represented as means ± SD. Data were processed as in panel B; no significant differences were found between colocalization indices. (D) Analysis of maximal mCherry-Wag31 (Che-Wag31, left column) and GFP-FAS-II fusion (GFP fusion, right column) fluorescence position within individual *Msm::mCherry-wag31* bacteria expressing each GFP fusion after 6 hours of induction. Each type of bacterium was scanned on both channels. Each dot represents the position of maximum fluorescence of the scans expressed in % of the highest values. Each dot was plotted against its position in the bacterium with the length of bacteria reported to 1. The names of the FAS-II proteins fused with GFP are indicated. Ø refers to the *Msm::mCherry-wag31* strain containing GFP alone.

Since FAS-II localization appeared to follow the reported localization of the polar protein Wag31 [29], [35], a recognized “old pole marker”, Wag31-FAS-II colocalization was therefore studied. For purposes of clarity, the word colocalization is used in this study to describe the localization of two proteins at the same focus but without implying the existence of any molecular interaction. A strain was constructed bearing a single but ectopic chromosomal copy of a mCherry-Wag31 fusion under the control of the constitutive promoter pHSP60 of the integrative vector pMV361. This type of construct, using the pHSP60 promoter, has been successfully used previously to study Wag31 localization [36]. When this strain was observed in fluorescence microscopy (Figure S3), the mCherry fusion was more visible at one pole as also commonly observed by others [36], [37]. This pole has been shown to represent the old pole (Figure 1) [29]. The fusions were introduced into the mCherry-Wag31 producing strain after which the fluorescence of both GFP and mCherry was analyzed as performed above on static images. As observed in the absence of mCherry-Wag31 (Figure 2A), GFP remained diffuse whereas GFP-MabA and GFP-InhA yielded intense polar foci, mainly at one pole (Figure 4A). GFP-KasA and GFP-KasB still appeared diffuse with a lower extend of polar spotting (Figure 4A). The overall localization pattern of the FAS-II fusions was comparable to that of the previous fusions (Figure 2A). The statistical relevance of the localization (Figure 4B) was analyzed as above on individual bacteria expressing each fusion (N) stemming from several individual slides (n>6). The polar indices of MabA (93.93±0.59 N = 800), KasA (23.87±4.56 N = 500) and KasB (13.49±1.43 N = 500) were not statistically different from the previously observed indices (see comparison between Figure 2B and 4B) whereas the PI of GFP-InhA (35.99±2.14 N = 700) was slightly reduced. While there was no dramatic effect of the presence of the mCherry-Wag31 fusion on the percentage of localization of the FAS-II proteins, there was the presence of colocalization of both Wag31 and FAS-II fusions foci. To quantify this phenomenon on a large number of bacteria a colocalization index was defined as being the percentage of bacteria exhibiting Wag31-GFP colocalization relative to the number of GFP-mCherry-positive bacteria. Colocalization indices (Figure 4C) were very high for all FAS-II proteins: MabA (83.31%±3.27, N = 800), InhA (93.93%±2.60, N = 700), KasA (89.71%±3.37, N = 500) and KasB (89.52%±3.60, N = 500). In order to evaluate the distribution of foci within individual bacteria, a more in-depth analysis was performed on a subset of bacteria by scanning more than 100 organisms of each type. On each scan (Figure 4D), the maximum fluorescence was plotted against the normalized length of the bacteria. A dot represented on a graph can refer to a focus (at the pole) or simply to the maximum of diffuse fluorescence along the cell. Localization of the reductases and colocalization with Wag31 at the old poles were clearly confirmed (Figure 4D). Furthermore, the maximum florescence emitted by KasA and KasB, which displayed the lowest polar indices, were clearly preferentially observed in proximity of the old poles along with Wag31, thus suggesting an actual preference for this localization (Figure 4D). The distribution of the polar foci clouds represented only about 20% of total cell size, i.e. less than 1 µm, probably due to light diffusion. This distribution was similar for FAS-II proteins and for Wag31, already known to be localized close to the polar membranes. In contrast, the maximum accumulation of GFP alone was never found at the old pole suggesting a polar exclusion of GFP alone. These analyses clearly demonstrate that the FAS-II proteins were preferentially located at the old pole with Wag31. Since Wag31 is acknowledged to be an old pole marker, we can conclude that the polar FAS-II proteins were located at the active growing “old pole” of the bacteria.

### FAS-II localization dynamics follow Wag31 dynamics with no molecular interaction

**Figure 5:**
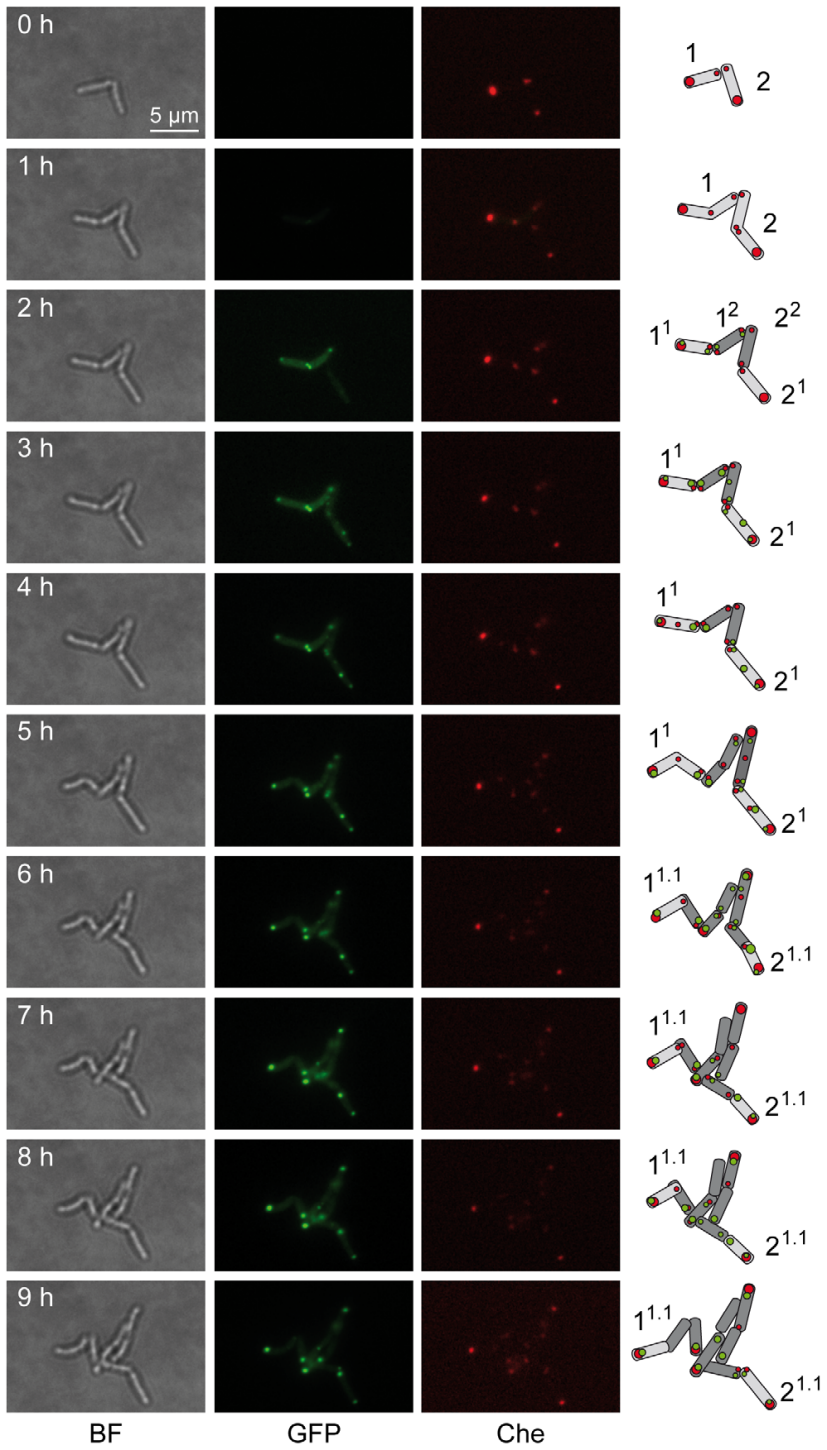
Dynamic of GFP-MabA/Wag31 colocalization. Images were extracted from the movie presented as movie S7. The time interval after the starting point of the movie is indicated in hours (h). Images were extracted from three channels (bright field, BF, left column; GFP, middle column; mCherry, Che, right column) in order to follow the dynamics of representative GFP-MabA and mCherry-Wag31 foci. Two bacteria, numbered 1 and 2, and their daughter cells, identified by superscript numbering are schematized on the right. Magnification: 630 X. Scale bar: 5 µm.

In order to compare FAS-II and Wag31 dynamics, time-lapse experiments were performed on bacteria expressing both types of fusions. The expression of mCherry-Wag31 being constitutive, polar Wag31 foci were visible in the mother cells at the beginning of the experiments. As observed on static images (Figure 4A), GFP alone was diffuse in the cytoplasm (Movie S6). In this movie, the presence of Wag31 at the old pole and its re-localization to the septum were clearly visible (Movie S6). In the GFP-FAS-II producing strains (Movies S7-S10), bacterial division was observed together with Wag31 and FAS-II dynamics. The dynamics of GFP foci were analyzed in detail for the GFP-MabA fusion (Movie S7 and Figure 5). Prior to the detection of GFP fluorescence, mCherry-Wag31 mainly observed at the old poles then, as the elongation progressed (1 h, bacteria 1 and 2), Wag31 was relocated to the septa as previously observed [29], [30]. The GFP fusions were first detected at these new poles and slightly at the old poles (2 h, bacteria 1^1^ and 1^2^). Progressively thereafter, establishment was observed at the old poles (see after 5 h, bacteria 1^1.1^). At the end of the experiments, GFP fluorescence was primarily observed at the old poles together with the brighter Wag31 fluorescence. Bacterium no. 2 presented here (Figure 5) displayed exactly the same dynamics with a delayed appearance at the beginning of the induction. Because of the lower expression of the InhA fusion, the dynamics of this fusion was more difficult to follow although the majority of GFP foci were established with the brighter Wag31 foci (Movie S8). Finally, the KasA fusion (Movie S9) and the KasB fusion (Movie S10) were mainly diffuse in the cytoplasm as already observed on static images and foci were observed only at the end of the experiments. When GFP fusion expression was induced, new proteins entered the system via the pole in construction (septal pole) and then established progressively to the old pole with Wag31.

To ascertain whether dynamic FAS-II-Wag31 colocalization was due, or not, to molecular interactions, *in vitro* Co-immunoprecipitation (Co-IP) experiments were performed with Wag31 against FAS-II proteins. *In vitro* ^35^S-labeled HA-tagged FAS-II proteins (h-proteins) bound to magnetic beads coated with anti-HA antibodies were used to trap labeled c-Myc-tagged Wag31 protein (c-Wag31). No Co-IP bands were observed, except with Wag31 itself (Figure S4A). When the Co-IP experiments were performed in the reverse direction (Figure S4B), with h-Wag31 against c-FAS-II proteins, only non-specific binding was observed with FAS-II proteins (Figure S4B, IPØ). This non-specific binding was not observed when known negative controls were used (Figure S4C) including human c-Lamin (c-Lam) or mycobacterial Nat protein (Arylamine N acetyltransferase, [38]). This indicates that FAS-II proteins localize to the old pole and follow Wag31 dynamics without any direct molecular interaction with Wag31.

### In vivo activity of GFP-fusions

**Figure 6:**
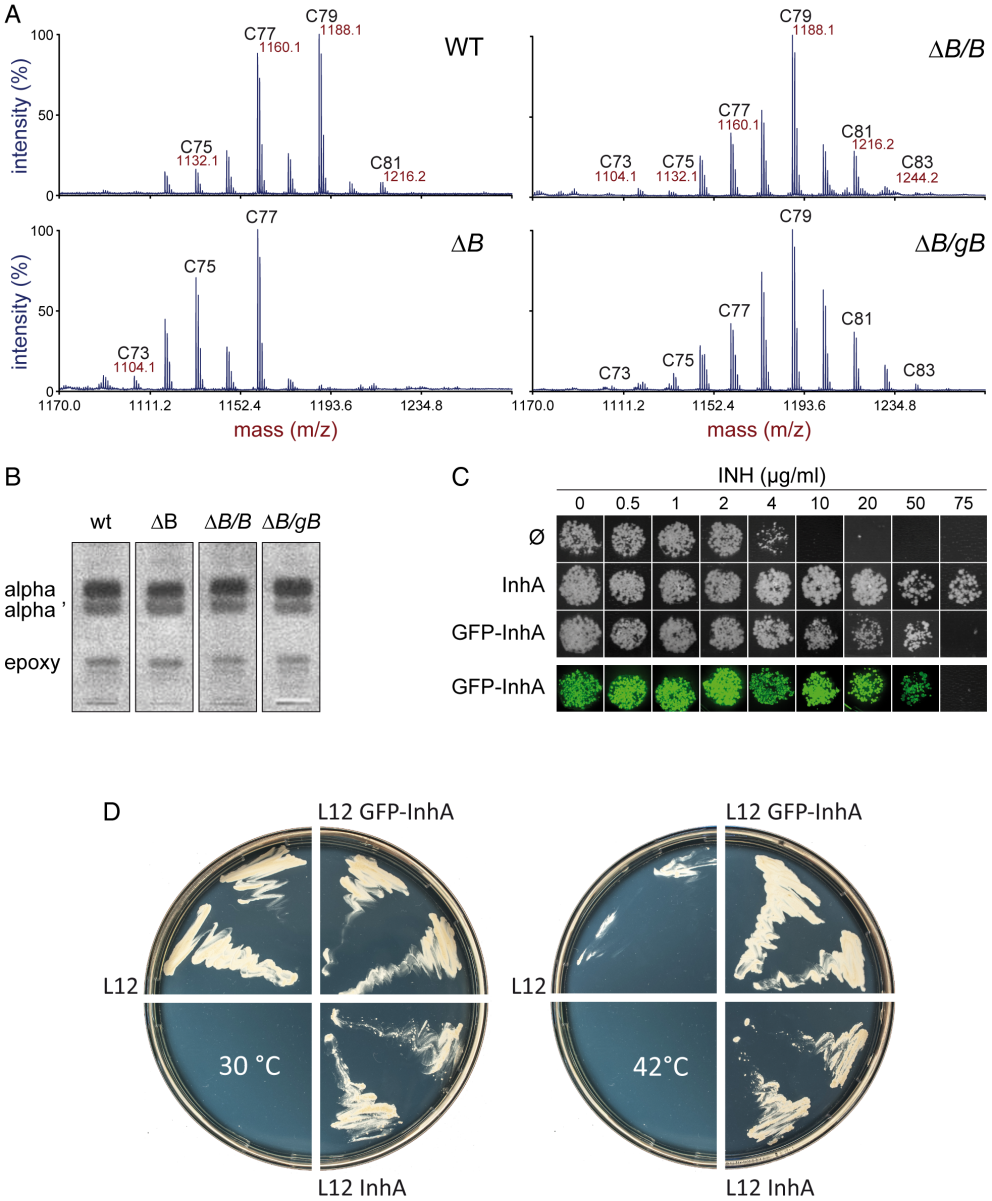
Analysis of GFP-KasB and GFP-InhA *in vivo* activities. (A) MALDI-TOF mass spectra of α-mycolic acid methyl esters extracted from wild type (*Msm* mc^2^155, WT), mutant (*Msm* mc^2^155 Δ*kasB*, Δ*B*) and complemented (*Msm* mc^2^155 Δ*kasB*/*kasB*, Δ*B/B*; *Msm* mc^2^155 Δ*kasB*/*gfp-kasB,* Δ*B/gB*) strains. The accurate masses (m/z), together with the length of the odd carbon-numbered mycolates are indicated. (B) TLC analysis of mycolic acid methyl esters extracted from the strains analyzed and described in Figure? 6A. The migration position of the α-mycolates (alpha), the α'-mycolates (alpha') and of the epoxy-mycolates (epoxy) are indicated. (C) Growth analysis of colonies of *Msm* mc^2^155 derivatives spotted onto agar plates containing increasing amounts of isoniazid (INH). Bacterial colonies were photographed either under white light (first three rows) or under blue light illumination allowing to reveal GFP fluorescence (last row). Bacteria containing the vector alone (Ø) or expressing the *Mtb inhA* gene, or the GFP fusion with InhA are represented. (D) Two colonies of the thermosensitive strain *Msm inhA* ^ts^ containing either the pLAM12 vector (L12), the pLAM12::GFP-InhA plasmid (L12 GFP-InhA) or the pLAM12::InhA plasmid (L12 InhA) were grown at 30°C (left) or 42°C (right) on agar plates containing acetamide (0.2%).

FAS-II proteins are organized in specialized complexes (Figure S2) interconnected with one another [16], [17], [18]. We showed above that representative members of theses complexes were located at the old poles during elongation and at the septa prior to division. It was thus tempting to postulate that the biosynthesis of mycolic acids may occur at these precise foci. To date, there is no known biochemical clue for locating MA biosynthesis *in vivo*. Thus, to address this question, we chose to analyze GFP-fusion activity *in vivo*. The *kasB* gene is the only FAS-II gene that is not essential in both *M. marinum* [39] and *Mtb* [40]. In the absence of KasB, which is part of the E2-FAS-II complex [17] and is responsible for the final step of meromycolate elongation, the MA are shortened by two to four carbons [40]. For the present purposes, we constructed a *kasB*-KO mutant of *Msm* (Figure S5) that was viable, indicating that *kasB* is also “non-essential” in *Msm*. In this mutant, the alpha- and epoxy-MA were shortened by four carbons (Figure 6A, 6B) whereas the alpha'-MA, which appeared as KasB independent, were not affected (Figure S6). The mutant phenotype was reverted by complementation with the wild type *kasB* gene and, more importantly, by the GFP-KasB fusion (Figure 6A). The localization pattern of GFP-KasB in the complemented mutant was identical to that observed with the wild type (data not shown). The GFP-KasB fusion was active in vivo.

Given that the other FAS-II genes (*mabA*, *inhA*, *kasA*) are essential [41], [42] and part of operons, it was not easily achievable, in our system, to test the GFP-fusion complementation of interrupted genes. *MabA* and *kasA* genes exist within operons whereas *inhA* lies at the end of an operon. Nevertheless, we had some inkling with regard to InhA. InhA is the primary INH (isoniazid, also known as isonicotinylhydrazine) target and its overproduction induces an increase in the level of INH resistance by trapping the INH-NAD adducts in its catalytic pocket, thus reducing INH concentration within the bacteria [43]. We first verified that the overproduction of the wild type *inhA* gene in our strain indeed increased INH resistance (18.75 fold; 75 µg/ml, Figure 6C) whereas the production of GFP alone did not affect this level (4 µg/ml). A clear increase in resistance threshold, (12.5 fold; 50 µg/ml), was also observed when GFP-InhA was produced. The GFP-InhA fusion was likely able to bind the INH-NAD adducts *in vivo* thereby inducing an increase in cell resistance to INH. Finally, to demonstrate that the GFP-InhA fusion was active, the complementation of an *inhA* thermosensitive mutant *Msm* strain (mc^2^2359; *inhA* ^ts^; [44]) was tested (Figure 6D). At 42°C, in the presence of acetamide, the *inhA* ^ts^ strain containing the void vector pLAM12 was unable to grow. In contrast, when either the wild type InhA protein or, more interestingly, the GFP-InhA fusion was expressed, the complemented strains were thermo-resistant indicating that the fusion was also able to complement the InhA deficiency. These results demonstrate that the GFP-InhA as well as the GFP-KasB fusion may be active *in vivo* while forming foci.

### Localization of the Mycolic acid export machinery

**Figure 7:**
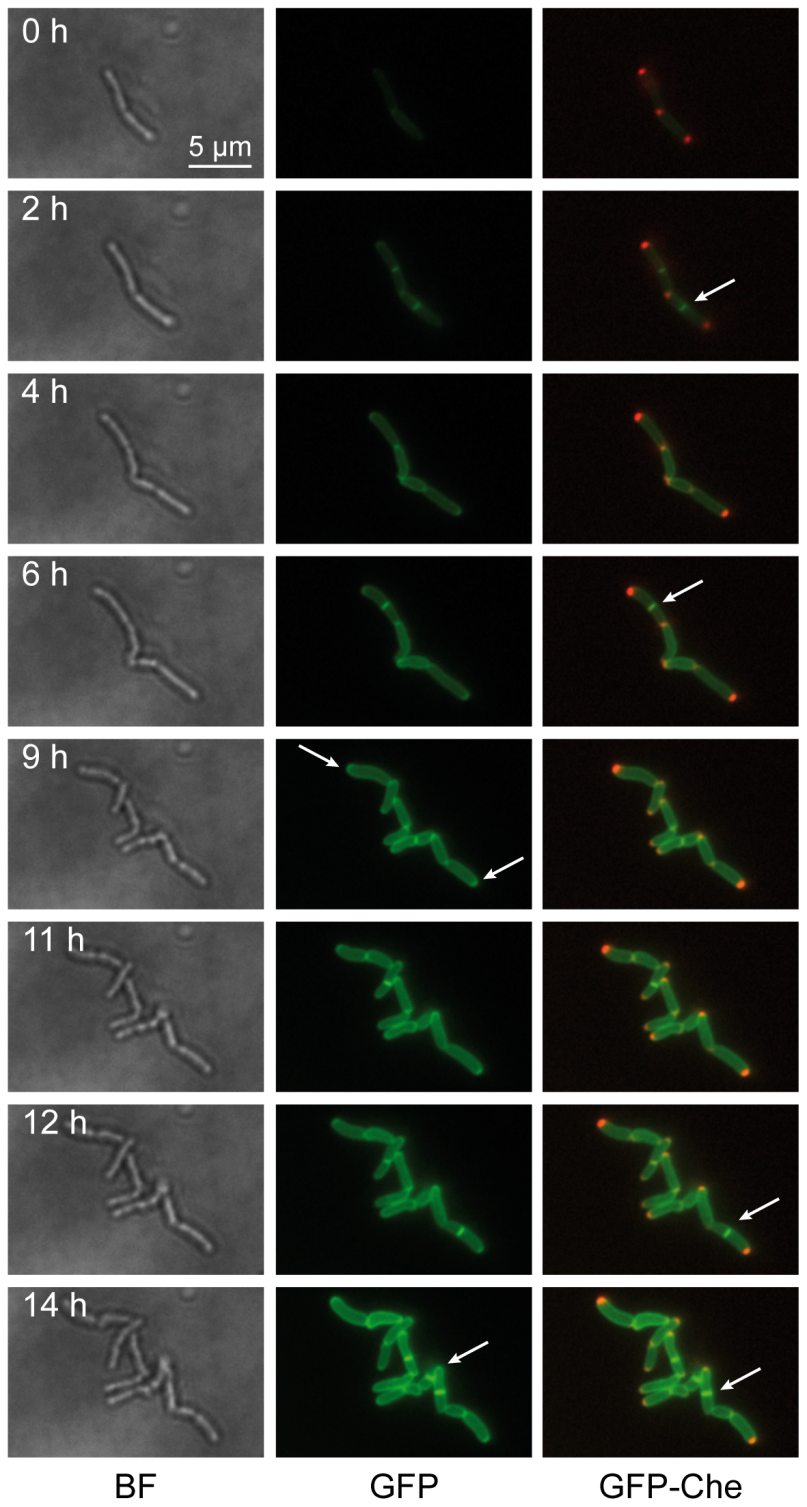
Dynamic localization of MmpL3. Images were extracted from the movie presented as movie S12. The time interval after the starting point of the movie is indicated in hours (h). Images were extracted from three channels (bright field, BF, left column; GFP, middle column; GFP and mCherry, GFP-Che, right column). The dynamics of the localization of the GFP fusions was followed in *Msm*::*mCherry-wag31* bacteria expressing the MmpL3-GFP fusions. The presence of MmpL3 in septa is indicated by white arrows in the right column. The “croissant”-shaped accumulation of MmpL3 at the poles is indicated by white arrows in the middle column. The position of mCherry-Wag31 at the poles and septa is clearly visible. Magnification: 630 X. Scale bar: 5 µm).

**Figure 8:**
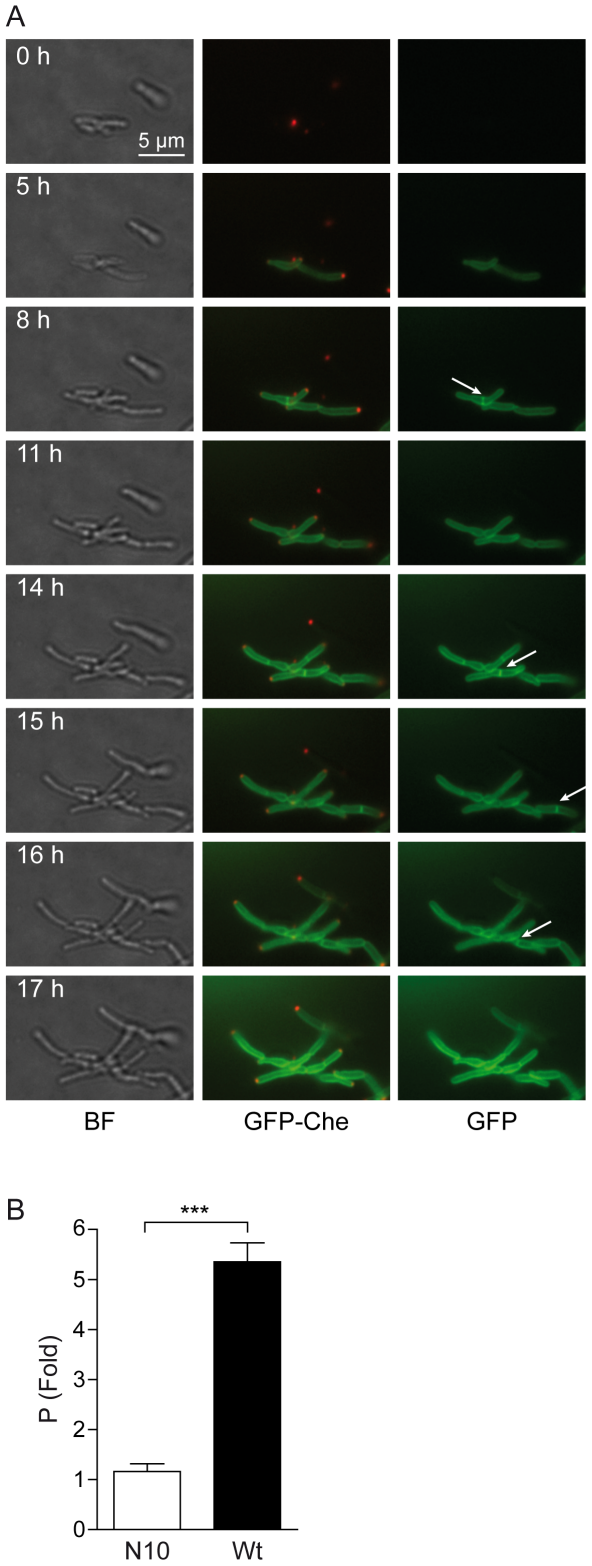
Dynamic localization of MmpL3-N10. (A) Images were extracted from the movie presented as movie S13. The time interval after the starting point of the movie is indicated in hours (h). Images were extracted from three channels (bright field, BF, left column; GFP and mCherry, GFP-Che, middle column; GFP, right column). The dynamics of the localization of the GFP fusions was followed in *Msm*::*mCherry-wag31* bacteria expressing the MmpL3-N10-GFP fusions. The presence of MmpL3-N10 in septa is indicated by white arrows in the right column. The position of mCherry-Wag31 at the poles and septa is clearly visible. Magnification: 630 X. Scale bar: 5 µm. (B) Bar graph representation of the localization of MmpL3 and MmpL3-N10 in *Msm* strains expressing each GFP fusion after 6 hours of induction. Data are represented as mean ± SEM. Data were collected from wide-field microscopy images (as in Figure? 7 and Figure? 8A) by scanning individual bacteria at the level of the membrane, the cytoplasm, and the poles as described in the Materials and Methods. The y-axis represents the polar accumulation (P) of either the complete (Wt) or the N-terminal portion (N10) of the MmpL3 transporter. A statistical *t*-test was performed to determine the difference between the polar accumulation of MmpL3-GFP (black bar, P = 5.353±0.3810 N = 192) and the absence of polar accumulation of MmpL3-N10-GFP (white bar, P = 1.160±0.1572 N = 113), with N indicating the number of bacteria analyzed. The unpaired *t*-test *p* value is symbolized by asterisks (***, *p*<0.0001).

Recently, the potential membrane protein MmpL3 has been identified in *Mtb* as an MA transporter [19], [45]. It is involved in the transport of TMM to the external mycomembrane. Because of the localization of major components of FAS-II observed herein, we hypothesized that MA may be synthesized at these *loci* to be subsequently transported at the actively growing envelope regions. GFP was therefore merged to the MmpL3 C-terminal given that this domain was predicted to be intra-cytoplasmic (Toppred2, [46], http://bioweb.pasteur.fr/seqanal/interfaces/toppred.html). MmpL3, a RND family protein [47], was predicted to be organized in twelve transmembrane helices. The localization of this fusion was analyzed as above. Upon performing time-lapse experiments with MmpL3-GFP alone (Movie S11), or in the presence of mCherry-Wag31 (Figure 7, extracted from Movie S12), a very clear envelope staining was observed together with preferential labeling of one pole and a very bright and flashing labeling of the septum, likely due to the presence of the double membrane at this position. The first conclusion was that MmpL3 might indeed be in the membrane or at least, in the envelope. Its C-terminal domain, fused with the GFP, could be intra-cytoplasmic because GFP is not fluorescent when it is located in another compartment [48]. As a localization control, a GFP fusion of the first ten transmembrane helices of MmpL3 (MmpL3-N10) was constructed and its localization analyzed *in vivo* as described above. Upon performing time-lapse experiments with MmpL3-N10-GFP in the presence of mCherry-Wag31 (Figure 8, extracted from Movie S13), the preferential MmpL3 polar localization was not observed. MmpL3-N10, which remained in the membrane, or at least in the envelope, was no more accumulated at the pole. The polar accumulation (P) of MmpL3 was quantified as described in Materials and Methods and compared to that of MmpL3-N10 (Figure 8B). MmpL3-N10 localization was not polar (P = 1.16 ± 0.15 N = 113) whereas MmpL3 was enriched by more than 5 fold at the pole (P = 5.35±0.38 N = 192).

The aspect of the images of the polar localization of MmpL3 differed significantly from that observed with the FAS-II components. The former was “crescent-shaped”, suggesting a membrane localization of MmpL3. MmpL3 was located in the envelope, probably in the cytoplasmic membrane, and was preferentially located at the growing bacterial tips containing the larger amounts of Wag31. In addition, MmpL3 was visible very early in the septa, likely before the appearance of Wag31 and membrane curvature. This polar localization of the MA transporter appeared to be due to a MmpL3 domain located between the end of helix 10 and the C-terminal end of the protein.

## Discussion

The main objective of the present study was to determine whether the biogenesis of mycolic acids is coordinated or not with bacterial envelope elongation. Through the use of a mycobacterial model of *Mtb*, *M. smegmatis*, we were able to localize MA biosynthesis FAS-II proteins *in vivo* and compare their localization dynamics to that of the polar protein Wag31. Wag31 is a main component of mycobacterial elongation and division [29], [37] and has been found to be associated with the curved membranes of old poles as well as of the division septum [49]. Herein; proteins of the FAS-II complex core [16], [17], [18] were observed to form foci associated with both the old poles and septal poles. While the FAS-II condensing enzymes were mainly diffuse, they were also able to form foci at the same locations. In dynamic studies, newly-synthesized ectopic FAS-II proteins were first located at the septal region suggesting that they may participate very early in pole construction. Thereafter, they were established rapidly at the old poles together with polar Wag31 foci [29]. Such polar localization of FAS-II proteins as well as their dynamic localization to the old poles along with Wag31 suggest the existence of a spatiotemporal coordination between MA biosynthesis and Wag31-controlled polar PG synthesis.

A key question was to determine whether foci, which were observed herein in bacteria expressing ectopic GFP fusions, correspond to actual complexes or to protein aggregates or inclusion bodies (IB). In rod-shaped bacterium *E.coli*, which does not display the same polar cell-wall elongation process as mycobacteria, the poles are not the active sites for PG synthesis [22]. In *E.coli*, the poles and the regions between the chromosomes are the areas where protein aggregates accumulate [50]. Furthermore, these foci do not separate after division and are preferentially accumulated in the mother cell during cellular aging [51]. In contrast, in mycobacteria, poles are very active loci where lateral PG synthesis [22] and most likely free MA synthesis [52], occur during bacterial elongation. In addition, poles are also involved in protein export [53], DNA transfer [32], chromosomal partitioning and DNA replication [54], [55] as well as phosphorylation by Ser/Thr protein kinases [56]. Mycobacterial poles are thus certainly not suitable for detrimental protein accumulation. A second argument against polar IB formation is the fact that asymmetric FAS-II polar localization was observed in the present model. *Actinobacteria* exhibit apical (polar) growth [24] and the distribution of polar proteins involved in PG biosynthesis is asymmetric with a preference for old poles [29]. Even though old pole-containing mycobacteria do not grow faster [57], polar growth itself appears to be asymmetric with the old pole being more active than the new pole [31]. Indeed, we observed herein a localization preference of FAS-II proteins to the old pole containing Wag31 whereas GFP alone seemed excluded from these poles. The foci clouds of FAS-II and Wag31 were located at the same position in the bacteria, at the very end of the organism. As commonly observed in *E.coli*, inclusion bodies are otherwise more likely to be found further inside the bacterial tips. Finally, three additional observations in the present study advocate against IB formation. First, there was no quantitative correlation between foci formation and GFP-fusion expression levels. Secondly, two of the four fusion proteins tested (KasB and InhA) were active in vivo while forming foci. Thirdly, during the division process, we observed foci splitting which can be compared to the active polarisome splitting in *Streptomyces* [58]. These finding strongly suggest that the FAS-II foci revealed the presence of FAS-II complexes mainly at the old poles and septal poles.

Mycolic acids are believed to be synthesized in the cytoplasm and transported to the periplasm as TMM by the trans-membrane RND-family transporter MmpL3 [19]. As expected, in view of its secondary structure and function, MmpL3 was observed at the level of the mycobacterial envelope. It is likely inserted in the plasma membrane similarly to that proposed in the original MmpL3 study [19]. Moreover, RND family proteins such as gram negative efflux pumps for example are inserted in the inner membrane and driven by the proton motive force [59] solely present at this level of the envelope. In addition, the MmpL3 C-terminal GFP fusion used herein was fluorescent, indicating that the C-terminus domain of MmpL3 is probably located in the cytoplasm. GFP functions as an efficient reporter for analysis of protein topology since it is fluorescent when located in the cytoplasm and non-fluorescent when transported outside the plasma membrane [48]. MmpL3-GFP was clearly more abundant in the membrane of Wag31-containing old poles while displaying a crescent-shaped fluorescence. This typical shape is similar to that observed for DivIVA, an ortholog of Wag31 in *B.subtilis* [28]. Moreover, this shape was very different from the shape of the GFP-FAS-II foci, suggesting that FAS-II was not inserted in the membrane as in the case for MmpL3 or occupying the inside of the membrane as in the case of Wag31. Finally, the most impressive images were obtained when the bacteria formed a septum: MmpL3 yielded a very intense labeling at the position of the septum probably due to its insertion into the double membrane of the septum. This MmpL3 localization pattern strongly resembled that reported for membrane Ser/Thr protein kinase PknB [56]. Indeed, Wag31 has been shown to be regulated by PknA and PknB [29]. PknA and B have been involved in the inhibition of many MA biosynthesis proteins such as KasA [60], mtFabH [61], MabA [62], InhA [63], [64] and the methyltransferase PcaA [65]. This suggests the existence of a common regulatory network between FAS-II, Wag31 and the cell wall elongation process. From the present data and in view of the literature, we can propose that mycolic acids are probably primarily exported at the poles and septa in proximity to Wag31 and FAS-II foci and at the *locus* of PG biosynthesis. The disappearance of the polar accumulation of MmpL3 upon deletion of its C-terminal end suggests that this region is probably involved in its localization. It also suggests that MmpL3 may be localized through a diffusion-capture mechanism using its C-terminal end as signal [66].

A puzzling question was to ascertain whether the polar localizations of FAS-II and MmpL3 reflect MA biosynthesis and export localization. Some reports can indeed be interpreted in this manner when compared with our results. *Mtb* polar lysis has been observed in very early EM images of isoniazid-treated wild-type *bacilli* [67]. Isoniazid targets InhA and if we assume that synthesis and export of mycolic acids are primarily polar, InhA inhibition will leave “holes” in the polar mycomembrane and ultimatly induce specific unipolar lysis as previously reported [67]. More recently, polar lysis was also observed when arabinan synthesis was inhibited with benzothiazinone [31], [52]. Finally, a link with MA synthesis was also attributed following MmpL3 localization. Because MmpL3 is accumulated at the pole, MA export (and probably MA synthesis) can be polar. By using unnatural trehalose analogs, two separate reports have shown that Ag85 activity, the mycoloyl-transferase that links mycolic acids to arabinogalactan, is concentrated in the envelope and at the poles of mycobacteria with a pattern strongly resembling that of MmpL3 herein [68], [69]. The linking of mycolic acids to the cell wall may occur mainly at these positions. As suggested by the present results, the C-terminal domain of MmpL3 may be involved in this localization.

In conclusion, the polar localization of external membrane lipid biosynthesis enzymes observed in the present study constitutes a novel observation in bacteria and *a fortiori* in mycobacteria. Moreover, the observation of the dynamic localization of the MA transporter MmpL3 strongly reinforces the possibility of a positioning of MA biosynthesis in these regions. In keeping with the existing literature, the present data further suggest the existence of the polar localization of complete MAPc synthesis and its coordination with bacterial cell elongation. Peptidoglycan is already known to be synthesized at these positions [70] as is likely arabinan [52]. Moreover, mycolic acids are linked to arabinogalactan at these positions [68], [69]. Present findings also show that FAS-II proteins, MmpL3 and Wag31 are present in the same region, at the same time in the bacteria; furthermore, it has recently been demonstrated that INH can affect the coupling of division and elongation [57]. Altogether, all the above data further place the pieces of the puzzle together and support the proposed model of localized envelope biogenesis machinery coordinated with the process of cell elongation and division. This coordinated process may ultimately represent the Achilles' heel of the pathogenic bacterium *Mycobacterium tuberculosis*.

## Materials and Methods

### Strains and culture conditions

Plasmid constructions were performed in the *Escherichia coli* K12 derivative Top10-F' (Invitrogen) using classical cloning procedures according to enzyme and product manufacturers' recommendations (NEBiolabs; Fermentas; Promega,). Culture media (Luria-Bertani (LB) broth, or LB-Agar) were supplemented when needed with either kanamycin (50 µg/mL), hygromycin-B (200 µg/mL), or chloramphenicol (12.5 µg/mL). *Mycobacterium smegmatis* mc^2^155 (ATCC 700084) was cultured in Middlebrook based media (Difco). Liquid cultures were grown in Middlebrook 7H9 (0.05% Tween 80, 10% ADC (BBL)) whereas bacteria were plated on Middlebrook 7H10 agar (10% OADC (BBL)). *Msm inhA* ^ts^ (mc^2^ 2359; [44]) is a derivative of *Msm* mc^2^155 bearing a punctual mutation in the *inhA* gene rendering both the protein and the strain thermosensitive. When needed, media were supplemented with either kanamycin (50 µg/mL), hygromycin-B (150 µg/mL) or zeocin (10 µg/ml).

### Plasmid constructions

The four *Mtb* FAS-II genes (*inhA, rv1484; mabA, rv1483; kasA, rv2245; kasB, rv2246*) were isolated from the previously described pGAD-T7::*fas-II* and pGBK-T7::*fas-II* derivatives [17] as either *Nde*1-*Bam*H1 (*kasA* and *mabA*), *Nde*1-*Eco*R1 (*inhA*), or *Nde*1-*Msc*1 fragments (*kasB*). They were cloned under the control of the acetamidase promoter (P*_amiE_*) at the corresponding sites of the inducible vector pLAM12 [71] to produce the pLAM12::*fas-II* derivatives (Table S1). For the construction of the fluorescence control vectors, the *egfp* and the *mCherry* genes were amplified respectively from pEGFPN1 (Clontech) and from pmCherry-N1 (Clontech) using specific pairs of primers creating a *Nde*1 site at the ATG position of each gene and a second site (*egfp*, *Hind*III; *mcherry*, *Bam*H1) at the end of each gene (Table S2). These genes were then cloned at the same sites in pLAM12 to yield pLAM12::*gfp* and pLAM12::*mCherry* (Table S1). For construction of N-terminal GFP fusions with the FAS-II proteins, the *egfp* gene was amplified from the pEGFPN1 vector using a second set of primers creating in-phase *Nde*1 sites both at the ATG and STOP positions (Table S2). The *egfp* gene was inserted at the *Nde*1 sites of each of the four FAS-II genes in the pLAM12 derivatives. For construction of the Wag31 fusion, the *wag31* gene (*rv2145c*) was amplified by PCR from *Mtb* H37Rv genomic DNA using specific primers (Table S2) creating a Nde1 site at the ATG position and cloned as an *Nde*1-*Bam*H1 fragment under the control of the pHSP60 promoter of the replicative vector pMV261-Nde [17], a derivative of pMV261 [72]. Using the same strategy as above, the *mCherry* gene was cloned, as an intermediate, at the *Nde*1 site of pMV261::*wag31* to yield a N-terminal fusion with Wag31. The fusion was further transported at an equivalent position of the integrative vector pMV361 [72]. The same wag31 *Nde*1-*Bam*H1 DNA fragment was also cloned at the equivalent positions of the Matchmaker III yeast two-hybrid cloning vectors pGAD-T7 and pGBK-T7 (Clontech) to produce pGAD-T7::*wag31* and pGBK-T7::*wag31*. In the same manner, the *Mtb nat* gene (*rv3566*) was cloned in the Matchmaker III vectors as *Nde*1-*Bam*H1 fragments. Finally, the *mmpL3* gene (*rv0206c*) and its N-terminal end containing only the first ten trans-membrane helices (Mp3-N10) were cloned at the *Nde*1 site of pLAM12::*gfp* to construct the C-terminal MmpL3-GFP and MmpL3-N10-GFP fusion plasmids pLAM12::*mmpL3::gfp* and pLAM12*::mp3-N10::gfp.* All constructs were verified by restriction analysis and sequencing (Millegen).

### Analysis of GFP-fusion expression in vivo

Msm liquid cultures were induced with 0.2% acetamide in early log phase (OD_590nm_ = 0.5) and samples were collected every two hours. Fluorescence at 525 nm (excitation at 485 nm) and OD_590nm_ were measured in micro-titration plates (Optiplate-96 well) with a FLx800 fluorescence reader (Biotek Instruments). Acquisition of fluorescence in the sample (F(s)) was performed in triplicate on serial dilutions of each culture. The data, expressed in relative fluorescence units (RLU), were corrected for the variation in fluorescence of the medium (F(m)) and the background fluorescence of the Msm/pLAM12 control strain (F(c)). Fluorescence (F) was also reported to the OD of the sample (OD(s)) and of the control strain (OD(c)) using the following formula: F  =  [(F(s) – F(m))/OD(s))]/[(F(c) – F(m))/(OD(c)]. The means ± SD (standard deviation) of the data were plotted against the time of induction using Graphpad Prism 5 software (GraphPad Software Inc.). The same culture samples were analyzed by Western blotting during the course of induction. Aliquots of known and equivalent OD were lysed, fractionated on 10% SDS-PAGE and appropriate amounts of total protein were blotted using mouse monoclonal anti-GFP (Roche Applied science ref: 11814460001) as primary antibodies. The blots were revealed by bioluminescence (ECL detection Kit, Millipore) and visualized by fluoroimaging.

### *In vitro* Co-immunoprecipitation (Co-IP)

*In vitro* transcription/translation of the genes of interest with the TnT Quick Coupled Transcription/Translation System (Promega) was performed as described previously [18] using supercoiled DNA from pGAD-T7 or pGBK-T7 derivatives expressing either the FAS-II genes [16], [17] or the *wag31 and nat* genes as substrates (Table S1). This system allowed generating L-[^35^S]-methionine-labeled proteins tagged with either the HA (hemagglutinin) epitope (pGAD-T7 derivatives) or the c-Myc epitope (pGBK-T7). The products were named h-proteins (HA-tagged) or c-proteins (c-Myc-tagged). For Co-IP experiments, the h-proteins and the c-proteins were mixed together with goat anti-mouse IgG magnetic beads (Dynabeads M-450) coated with monoclonal anti-HA antibodies (Sigma). Proteins ratios were adjusted to 1∶1 as described by assessing their specific activities (phosphorimaging) and correcting for their differences in the number of methionine residues. The co-IP reactions were performed as previously described [18] and analyses performed by separation on SDS-PAGE (4-20%) and phosphorimaging.

### Construction of the Mycobacterium smegmatis kasB knockout strain

The *Msm kasB* gene (*MSM*EG_4328) was disrupted by using the “recombineering method” developed by van Kessel and Hatfull [71]. The linear allelic exchange substrate (AES; Figure S5) was constructed using a three fragments fusion-PCR [73] with the indicated primers (Table S2). The AES was constituted, from 5′ to 3′, by the fusion of the 800 bps of DNA upstream from the initiator codon of *kasB*, followed by the Zeocin (InvivoGen) resistance gene (*Sh ble*,) from the pZeo vector [74] and ended by the 800 bps of DNA downstream from the stop codon of *kasB*. The substrate was subsequently introduced by electro-transformation into a recipient *Msm* strain already containing a hygromycin-resistant derivative of the recombineering plasmid pJV53 encoding for phage recombinases [71]. The construction and the selection of the mutant were essentially performed as previously described [71]. The chromosomal structure of the mutant (Figure S5) was verified by PCR with suitable primers (Table S2 and Figure. S5). After curing of the recombineering plasmid, the mutant strain was transformed by appropriate plasmid to produce the control strain (*Msm*-*ΔkasB*/pLAM12) and the complemented strains (*Msm*-*ΔkasB*/pLAM12::*kasB* and *Msm ΔkasB*/pLAM12::*gfp-kasB*)).

### Mycolic acid analysis

MA was performed essentially as described [75]. Bacterial residues obtained after lipid extraction with organic solvents were saponified at 110°C for 3 h in a mixture of KOH (40%) and methoxyethanol (C_3_H_8_O_2_) (1∶7, v/v). After acidification, fatty acids were extracted with diethyl ether and methylated with an ethereal solution of diazomethane. The mycolate patterns of the strains were determined by analytical high performance thin-layer chromatography (HPTLC) on Silica gel 60 (Merck) using dichloromethane 100% as eluent and revealed with rhodamine B (Sigma). The different classes of mycolates were separated by preparative TLC (Macherey-Nagel Silica Gel 60), using the same eluent. The lengths of the various types of purified mycolates were determined by MALDI-TOF mass spectrometry as previously described. MALDI mass spectra were acquired in reflectron mode using an Applied Biosystems 4700 Analyzer equipped with a Nd∶YAG laser (355 nm wavelength, <500-ps pulse and 200Hz repetition rate). The shots (2500 total) were accumulated in positive ion mode and MS data were acquired using the instrument default calibration. Mycolate samples were dissolved in chloroform at a concentration of 1 mM, and were directly spotted onto the target plate as 0.5 µl droplets, followed by the addition of 0.5 µl matrix solution. Samples were allowed to crystallize at room temperature. The matrix used was 2,5-dihydroxybenzoic acid (10 mg ml−1) in CHCl_3_/CH_3_OH (1 ∶ 1).

### Microscopy and video-microscopy

*Msm* liquid cultures expressing either the GFP protein fusions alone, or the GFP fusions together with the mCherry-Wag31 fusion were induced in log phase (OD_590nm_ 0.5) with 0.2% acetamide during 6 hours. Bacteria were pelleted, concentrated (20X) in PBS, and spread (10 µL) on poly-L-lysine coated glass slides (PolyScience). Slides were mounted with Dako mounting medium and observed with a large field Leica DMIRB fluorescence microscope at 63X magnification (Leica HCX Plan Apo x63/1.40-Oil). GFP (488 nm/509 nm) was revealed with a GFP-ET filter cube (excitation: BP 470/40; dichroic mirror, 495; emission: LP 525/50) whereas mCherry fluorescence (587 nm/610 nm) was visualized with a N2.1 Leica filter cube (excitation: BP 515–560; dichroic mirror, 580; emission LP 590). Images were captured with a CoolSnap HQ2 CCD camera (1 pixel = 6.45 µM), acquired with MetaMorph 7.1.7 software and processed with ImageJ software (National Institutes of Health, Bethesda, MD). All different images were acquired with the same exposure time (200 ms). Image processing consisted in equivalent adjustments of brightness and contrast on complete images. Gamma and LUT (Look Up Table) values were not modified and were left as linear on each channel. For statistical analysis and quantification of the polar indices, multiple slides (n) of independent experiments were used. An average of 1500 bacteria (N; see text) were counted from a minimum of three different slides and a maximum of 16 slides (3<n<16). Student *t*-tests were performed on raw data using the GraphPad Prism 5.0 software. Polar indices were expressed as percentages of polar foci in the fluorescent population and presented as mean with standard deviation (SD). For precise analysis of colocalization, each channel (GFP and mCherry) on microscopy images from individual bacteria producing both types of fusion (greater than 100) were scanned using the ROI manager of ImageJ software (NIH-USA). Thereafter, the GFP and the mCherry fluorescence profiles of each bacterium were obtained with the Plot Profile plugin. The maximum fluorescence levels of each scan of a given strain were normalized to the highest fluorescence value in this strain and plotted against their position along the long axis of the bacterium. The total length of each bacterium was reported to 1.

In order to quantify the polar localization of MmpL3, images from wide field fluorescence microscopy of *Msm* expressing either MmpL3-GFP or MmpL3-N10-GFP were analyzed with ImageJ software (NIH-USA) using the Plot Profile function. On each individual bacterium, three plots along 0.7 µm width lines were obtained: the first inside the cytoplasm (Background Fluorescence, F_BG_), the second along the long axis of the bacterium and passing through one pole (Polar Fluorescence, F_PO_), and the third along the short axis of the bacterium and crossing the membranes (Membrane fluorescence, F_MB_). On each bacterium, the maximum fluorescence (FMax) of each plot was collected. The analysis was performed on more than 100 individual bacteria. The polar localization fold ratio was calculated with the formula, [|F_PO_Max-F_BG_Max|]/[|F_MB_Max-F_BG_Max|], yielding a value of 1 when there was no polar localization and a value superior to 1 when the fluorescent protein was accumulated at the membrane pole. Polarization was expressed as mean ± SEM (Standard Error to the Mean), with N represent here the number of bacteria analyzed.

Time-lapse video microscopy was performed using an agar pad method essentially as previously described [34]. The bacteria were grown to mid-log phase, and aliquots (400 µL) were applied onto the coverslip of 35 mm glass bottom dishes (MatTek). After 5 minutes, the liquid was removed by aspiration and the bacteria were covered by 7H9 medium (10% ADC) at 37°C containing 0.6% Noble agar (Sigma), along with the appropriate antibiotics and the inducer (acetamide, 0.02%). After 1 hour solidification, the plates were pre-incubated for three hours at 37°C. The fluorescence was detectable in control GFP-producing bacteria. This time was chosen as the starting point (t = 0) for all time lapse experiments. Thereafter, the plates were observed as above using the Leica DMIRB florescence microscope equipped with a 37°C humid chamber. GFP and mCherry fluorescence was visualized with a GFP/mCherry-ET filter cube (Chroma Technology; excitation, no selection; dichroic filter, SP490-BP570/40; emission BP522/44-BP633/54). Individual frames (100 ms exposure) were acquired at regular time intervals (from 10 min to 2 hours) with MetaMorph 7.1.7 software during 14 to 20 hours. The representative movies were generated using ImageJ software. The required channels were extracted from the time-lapse image stacks and combined as described in the supplemental movie legends, after which they were converted to AVI (Audio Video Interleave) format at 0.5 to 1.5 frames per second using JPEG compression. Minimal image processing was performed as described above for static images.

## Supporting Information

Figure S1 **The mycolic acid biosynthesis pathway.** The substrates and products of the Fatty Acid Synthase of type I (FAS-I) and II (FAS-II) are indicated together with the biochemical reactions (black arrows) necessary to achieve the biosynthesis of mature mycolic acid (see [8] and [9] for review). The enzymes responsible for each reaction are identified in colored circles. The cofactors necessary for reduction reactions to occur (NADPH, NADH) were omitted for reasons of clarity. The proximal and distal positions of the meromycolic chain modifications by MA-Mtfs are indicated as P and D respectively. Intermediate reactions not detailed (acyl-chains activation) are symbolized by interrupted arrows. The synthesis of the meromycolic chain is initiated by the condensation by the mtFabH protein of the acyl-CoA products of FAS-I with malonyl-ACP by MtFabD to give a keto-acyl-ACP. After reduction by the keto-acyl-ACP reductase MabA, followed by dehydration by the hydroxyl-acyl-dehydratases HadAB or HadBC and reduction by the enoyl-ACP reductase InhA, the acyl-chain enters into a new cycle of elongation through condensation by the keto synthase KasA or KasB with a new malonyl-ACP unit. After its synthesis, the meromycolic chain is adenylated and ligated by FadD32 onto Pks13, which is the terminal condensing enzyme that links the meromycolic chain to a carboxylated alpha chain produced by FAS-I. The remaining keto function of the generated mycolic motif is then reduced by CmrA to yield the mycolic acid.
(TIF)

Figure S2 **The Mycolic Acid Biosynthesis Interactome.** The Mycolic Acid Biosynthesis Interactome (M.A.B.I.) is composed of three types of complexes: (i) the ‘initiation FAS-II’ (I-FAS-II) contains mtFabH interacting with a core (MabA, InhA and mtFabD) and represents the link between FAS-I and FAS-II; (ii) two ‘elongation FAS-II’ (E-FAS-II) complexes comprise the core interacting preferentially with either KasA and HadAB (E1-FAS-II) or KasB and HadBC (E2-FAS-II); these two complexes are thought to be capable of elongating acyl-AcpM to produce full-length meromycoloyl-AcpM, (iii) the ‘termination FAS-II’ (T-FAS-II) involves Pks13 interacting with KasB and is thought to condense the α-branch with the meromycolic branch. Our working hypothesis is that the formation of each complex type may be successive and that the acyl-ACP substrates may be channeled from one type of complex to another as a function of its elongation status. The FAS-II initiation complex (I-FAS-II), the elongation complexes 1 and 2 (E1-FAS-II and E2-FAS-II) and the termination complex (T-FAS-II) are represented in grey. The condensing enzymes KasA, KasB and MtFabH (FabH) are in orange. The interactions between the HadAB (AB) and HadBC (BC) dehydrase heterodimers (in green) with the MA-Mtf (in violet) are symbolized by curved arrows. The core (in yellow) symbolizes the reductases MabA and InhA together with the malonylCoA ACP transacylase MtFabD. The terminal condensing enzyme Pks13 is depicted in blue. The direction of trafficking of the elongating substrate is symbolized by arrows emanating from acyl-CoA (from FAS-I) toward the mature mycolic acid. Interrupted arrows symbolize omitted steps of substrate modifications.
(TIF)

Figure S3 **Localization of mCherry-Wag31 in** ***Msm::mcherry-wag31*** **.** Enlarged images of wide-field microscopy experiments on mCherry-Wag31 fusion-expressing bacteria as presented in Figure 2 and Figure 4. Brightfield images (BF; leftmost column) together with mCherry fluorescence images (Che, middle column) of *Msm* mc^2^ 155::pMV361::*mCherry* (Ø) and *Msm* mc^2^155::pMV361::*mCherry-wag31* (Wag31) were acquired 6 hours after dilution of the main culture and in the presence of anhydro-tetracyclin (50 ng/mL) for the latter. The merged images (right columns) allowed visualizing the localization of Che-Wag31 mainly at one pole of the bacteria (the old pole). Total optical magnification: 630 X. Scale bar: 5 µm.
(TIF)

Figure S4 **Analysis of FAS-II/Wag31 interactions by co-immunoprecipitation.** L-[^35^S]-methionine-labeled h-proteins (HA tagged; h-protein) and c-proteins (c-Myc tagged; c-protein) from *in vitro* transcription translation reactions as well as the Co-IP reaction products were fractionated by SDS-PAGE (4–20%) followed by phosphorimaging analysis. The gels containing the h-Wag31 protein alone or the c-Wag31 alone are represented in the leftmost lanes. The names of the c-proteins or h-proteins used in the Co-IP experiments are indicated above each panel. These proteins were run alone (-), or after Co-IP (IP). (**A**) c-Wag31 was tested against h-FAS-II. (**B**) h-Wag31 was tested against c-FAS-II proteins; IPØ refers to a Co-IP performed between a given c-protein and a void extract (obtained with the pGAD-T7 empty vector). (**C**) h-Wag31 was tested against human laminC (c-Lam) with *Mtb* Arylamine N-acetyltransferase (c-Nat) used as negative control. The black arrowheads represent a Co-IP band while the open arrowheads indicate the position of a missing Co-IP band corresponding to a negative interaction. When the sizes of both proteins were too close to each other, non-labeled h-proteins were used for Co-IP experiments as indicated with an asterisk.
(TIF)

Figure S5 **Construction and characterization of** ***M.smegmatis*** **Δ** ***kasB*** **.** (**A**) The wild type region (WT) of the *Msm* chromosome comprising *kasA* (open arrow), *kasB* (grey arrow) and *accD6* (open arrow) is depicted as a solid line together with the positions for hybridization of PCR primers (Wt-Fwd and Wt-Rev). The AES product synthesized by fusion-PCR is also presented with the primers used for its synthesis. The Zeocin resistance gene (*Sh ble*; *ble*) is represented as a black arrow. The structure of the chromosomal region after the allelic exchange is illustrated together with the positions of PCR primers used for its characterization. The lengths of each PCR product are indicated in base pairs (bps) and represented by dashed lines. (**B**) Ethydium bromide staining of an agarose gel of PCR products of wild-type (Wt) and *ΔkasB* (Δ) *Msm* genomic DNA. The primer pairs used for amplification are indicated above the gel. The migration positions of control linear DNA are indicated along with their respective sizes (in kbp).
(TIF)

Figure S6 **MALDI-TOF analysis of α'- and epoxy- mycolic acid methyl esters.** Mass spectra of α'-mycolic acid (left column) and epoxy-mycolic acid methyl esters (right column) extracted from wild type (*Msm* mc^2^155, WT), mutant (*Msm* mc^2^155 Δ*kasB*, Δ*B*) and complemented (*Msm* mc^2^155 Δ*kasB*/*kasB*, Δ*B/B*; *Msm* mc^2^155 Δ*kasB*/*gfp-kasB*, Δ*B/gB*) strains. The accurate masses (m/z), together with the length of the even carbon-numbered epoxy-mycolates and the odd carbon-numbered α'-mycolates are indicated.
(TIF)

Table S1 **Description of plasmids.**
(DOCX)

Table S2 **Oligonucleotide sequences of PCR primers.**
(DOCX)

Movie S1 **Localization of GFP.** Growth of *Msm* derivatives expressing GFP alone under the control of the p_amiE_ promoter was followed by fluorescence time lapse video-microscopy. The movie (left panel) obtained by merging the bright field channel (in red) with the GFP channel (in green) is presented with the movie (right panel) displaying only the GFP channel. AVI file (0.8 frames per second) was obtained as described in Materials and Methods at 0.8 frames per second with one frame per hour. Time intervals are indicated in hours (h). Scale bar: 5 µm.
(AVI)

Movie S2 **Localization of GFP-MabA.** Growth of *Msm* derivatives expressing the GFP-MabA fusion under the control of the p_amiE_ promoter was followed by fluorescence time lapse video-microscopy. Movie treatment is identical to that of Movie S1
(AVI)

Movie S3 **Localization of GFP-InhA.** Growth of *Msm* derivatives expressing the GFP-InhA fusion under the control of the p_amiE_ promoter was followed by fluorescence time lapse video-microscopy. Movie treatment is identical to that of Movie S1
(AVI)

Movie S4 **Localization of GFP-KasA.** Growth of *Msm* derivatives expressing the GFP-KasA fusion under the control of the p_amiE_ promoter was followed by fluorescence time lapse video-microscopy. Movie treatment is identical to that of Movie S1 except that the AVI file was obtained at 1.5 frames per second with one frame obtained every 15 min.
(AVI)

Movie S5 **Localization of GFP-KasB.** Growth of *Msm* derivatives expressing GFP-KasB alone under the control of the p_amiE_ promoter was followed by fluorescence time lapse video-microscopy. Movie treatment is identical to that of Movie S1
(AVI)

Movie S6 **Localization of mCherry-Wag31 and GFP.** Growth of *Msm::pMV361 mCherry-Wag31* derivatives expressing GFP alone under the control of the p_amiE_ promoter was followed by fluorescence time lapse video-microscopy. The movie (left panel) obtained by merging together the bright field channel (in blue), the GFP channel (in green) and the mCherry channel (in red) is presented with the movie displaying only the merged GFP and the mCherry channels (right panel). The AVI file was obtained as described in Materials and Methods at 0.5 frames per second. With one frame obtained every hour. The time intervals are indicated in hours (h). Scale bar: 5 µm.
(AVI)

Movie S7 **Localization of mCherry-Wag31 and GFP-MabA.** Growth of *Msm::pMV361 mCherry-Wag31* derivatives expressing the GFP-MabA fusion under the control of the p_amiE_ promoter was followed by fluorescence time lapse video-microscopy. Movie treatment is identical to that of Movie S6 except that the AVI file was obtained at 1.5 frames per second with one frame obtained every 10 min.
(AVI)

Movie S8 **Localization of mCherry-Wag31 and GFP-InhA.** Growth of *Msm::pMV361 mCherry-Wag31* derivatives expressing the GFP-InhA fusion under the control of the p_amiE_ promoter was followed by fluorescence time lapse video-microscopy. Movie treatment is identical to that of Movie S6 except that the AVI file was obtained at 1.5 frames per second with one frame obtained every 15 min.
(AVI)

Movie S9 **Localization of mCherry-Wag31 and GFP-KasA.** Growth of *Msm::pMV361 mCherry-Wag31* derivatives expressing the GFP-KasA fusion under the control of the p_amiE_ promoter was followed by fluorescence time lapse video-microscopy. Movie treatment is identical to that of Movie S6 except that the AVI file was obtained at 1.5 frames per second with one frame obtained every 15 min.
(AVI)

Movie S10 **Localization of mCherry-Wag31 and GFP-KasB.** Growth of *Msm::pMV361 mCherry-Wag31* derivatives expressing the GFP-KasB fusion under the control of the p_amiE_ promoter was followed by fluorescence time lapse video-microscopy. Movie treatment is identical to that of Movie S6.
(AVI)

Movie S11 **Localization of MmpL3-GFP.** Growth of *Msm* derivative expressing the MmpL3-GFP fusion under the control of the p_amiE_ promoter was followed by fluorescence time lapse video-microscopy. Movie treatment is identical to that of Movie S1
(AVI)

Movie S12 **Localization of mCherry-Wag31 and MmpL3-GFP.** Growth of *Msm::pMV361 mCherry-Wag31* derivatives expressing the MmpL3-GFP fusion under the control of the p_amiE_ promoter was followed by fluorescence time lapse video-microscopy. Movie treatment is identical to that of Movie S6.
(AVI)

Movie S13 **Localization of mCherry-Wag31 and MmpL3-N10-GFP.** Growth of *Msm::pMV361 mCherry-Wag31* derivatives expressing the GFP fusion of the membrane domain of MmpL3 (Mp3-N10-GFP) under the control of the p_amiE_ promoter was followed by fluorescence time lapse video-microscopy. Movie treatment is identical to that of Movie S6.
(AVI)
