# Supplementary figures and images for: Mycobacterium tuberculosis Proteins Involved in Mycolic Acid Synthesis and Transport Localize Dynamically to the Old Growing Pole and Septum
Source: PLoS One. 2014 May 9;9(5):e97148. doi: 10.1371/journal.pone.0097148 (PMC4016276; doi:10.1371/journal.pone.0097148)

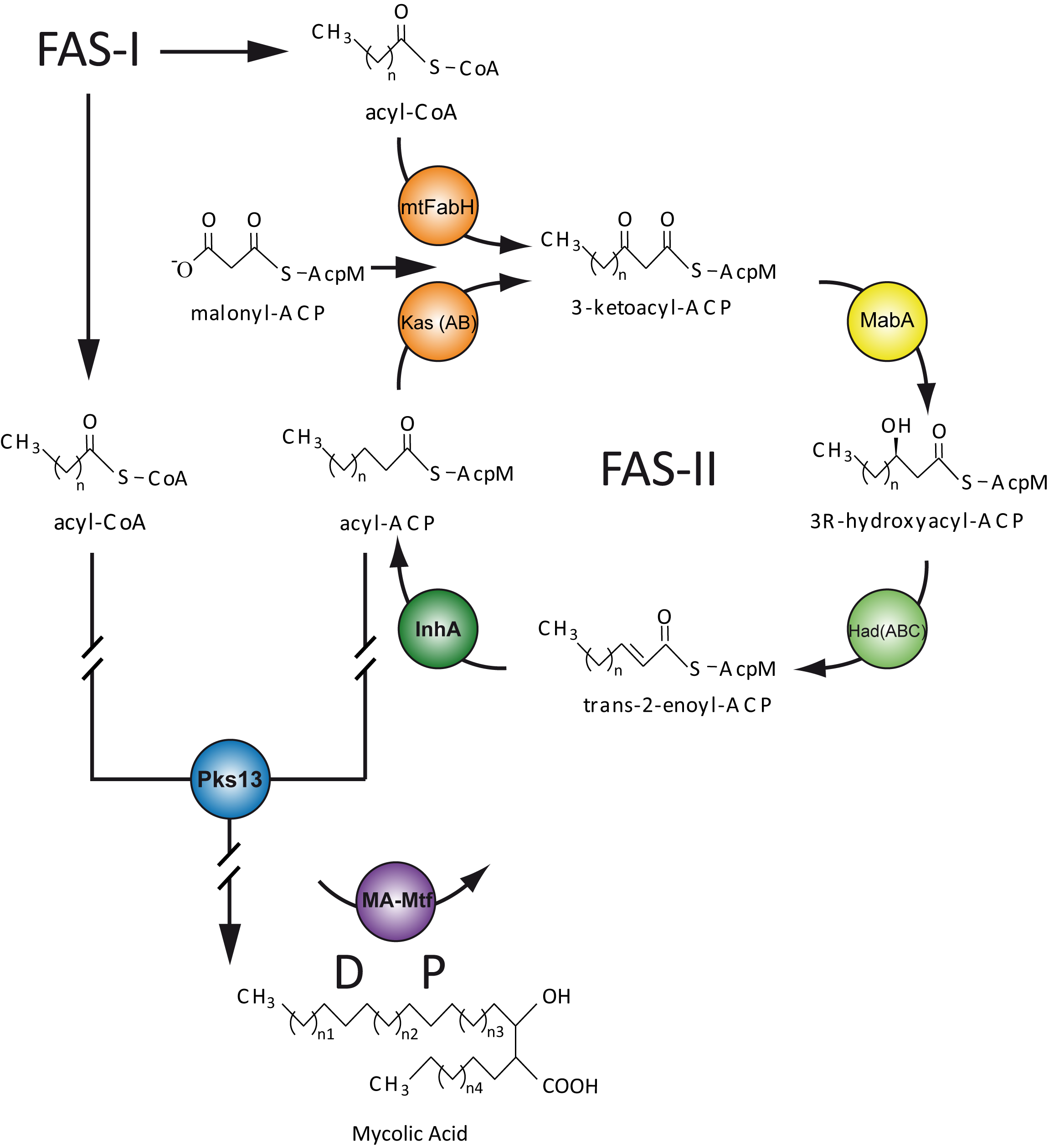

Supplement: Figure S1 — The mycolic acid biosynthesis pathway. The substrates and products of the Fatty Acid Synthase of type I (FAS-I) and II (FAS-II) are indicated together with the biochemical reactions (black arrows) necessary to achieve the biosynthesis of mature mycolic acid (see [8] and [9] for review). The enzymes responsible for each reaction are identified in colored circles. The cofactors necessary for reduction reactions to occur (NADPH, NADH) were omitted for reasons of clarity. The proximal and distal positions of the meromycolic chain modifications by MA-Mtfs are indicated as P and D respectively. Intermediate reactions not detailed (acyl-chains activation) are symbolized by interrupted arrows. The synthesis of the meromycolic chain is initiated by the condensation by the mtFabH protein of the acyl-CoA products of FAS-I with malonyl-ACP by MtFabD to give a keto-acyl-ACP. After reduction by the keto-acyl-ACP reductase MabA, followed by dehydration by the hydroxyl-acyl-dehydratases HadAB or HadBC and reduction by the enoyl-ACP reductase InhA, the acyl-chain enters into a new cycle of elongation through condensation by the keto synthase KasA or KasB with a new malonyl-ACP unit. After its synthesis, the meromycolic chain is adenylated and ligated by FadD32 onto Pks13, which is the terminal condensing enzyme that links the meromycolic chain to a carboxylated alpha chain produced by FAS-I. The remaining keto function of the generated mycolic motif is then reduced by CmrA to yield the mycolic acid. (TIF) [file pone.0097148.s001.tif]

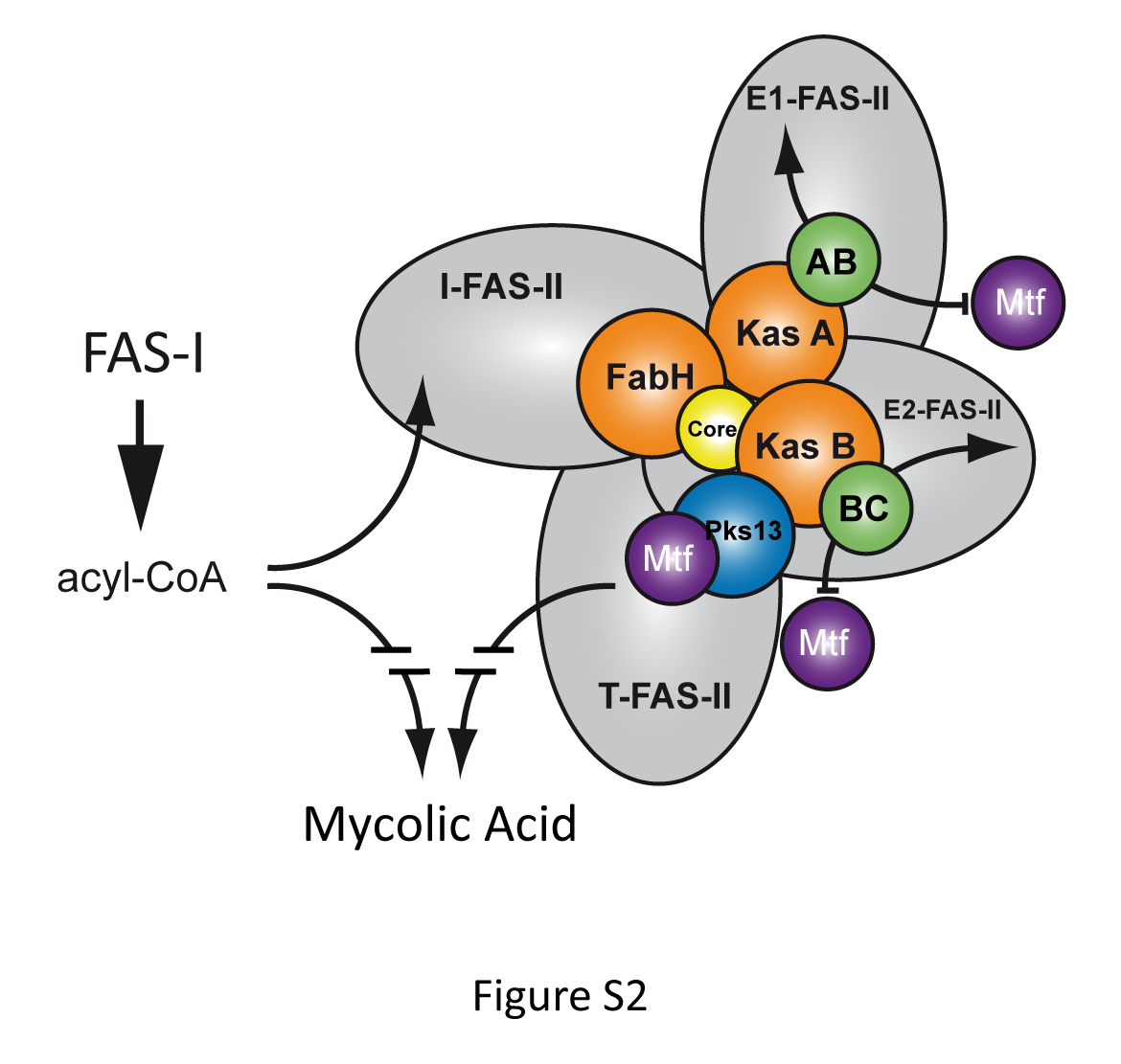

Supplement: Figure S2 — The Mycolic Acid Biosynthesis Interactome. The Mycolic Acid Biosynthesis Interactome (M.A.B.I.) is composed of three types of complexes: (i) the ‘initiation FAS-II’ (I-FAS-II) contains mtFabH interacting with a core (MabA, InhA and mtFabD) and represents the link between FAS-I and FAS-II; (ii) two ‘elongation FAS-II’ (E-FAS-II) complexes comprise the core interacting preferentially with either KasA and HadAB (E1-FAS-II) or KasB and HadBC (E2-FAS-II); these two complexes are thought to be capable of elongating acyl-AcpM to produce full-length meromycoloyl-AcpM, (iii) the ‘termination FAS-II’ (T-FAS-II) involves Pks13 interacting with KasB and is thought to condense the α-branch with the meromycolic branch. Our working hypothesis is that the formation of each complex type may be successive and that the acyl-ACP substrates may be channeled from one type of complex to another as a function of its elongation status. The FAS-II initiation complex (I-FAS-II), the elongation complexes 1 and 2 (E1-FAS-II and E2-FAS-II) and the termination complex (T-FAS-II) are represented in grey. The condensing enzymes KasA, KasB and MtFabH (FabH) are in orange. The interactions between the HadAB (AB) and HadBC (BC) dehydrase heterodimers (in green) with the MA-Mtf (in violet) are symbolized by curved arrows. The core (in yellow) symbolizes the reductases MabA and InhA together with the malonylCoA ACP transacylase MtFabD. The terminal condensing enzyme Pks13 is depicted in blue. The direction of trafficking of the elongating substrate is symbolized by arrows emanating from acyl-CoA (from FAS-I) toward the mature mycolic acid. Interrupted arrows symbolize omitted steps of substrate modifications. (TIF) [file pone.0097148.s002.tif]

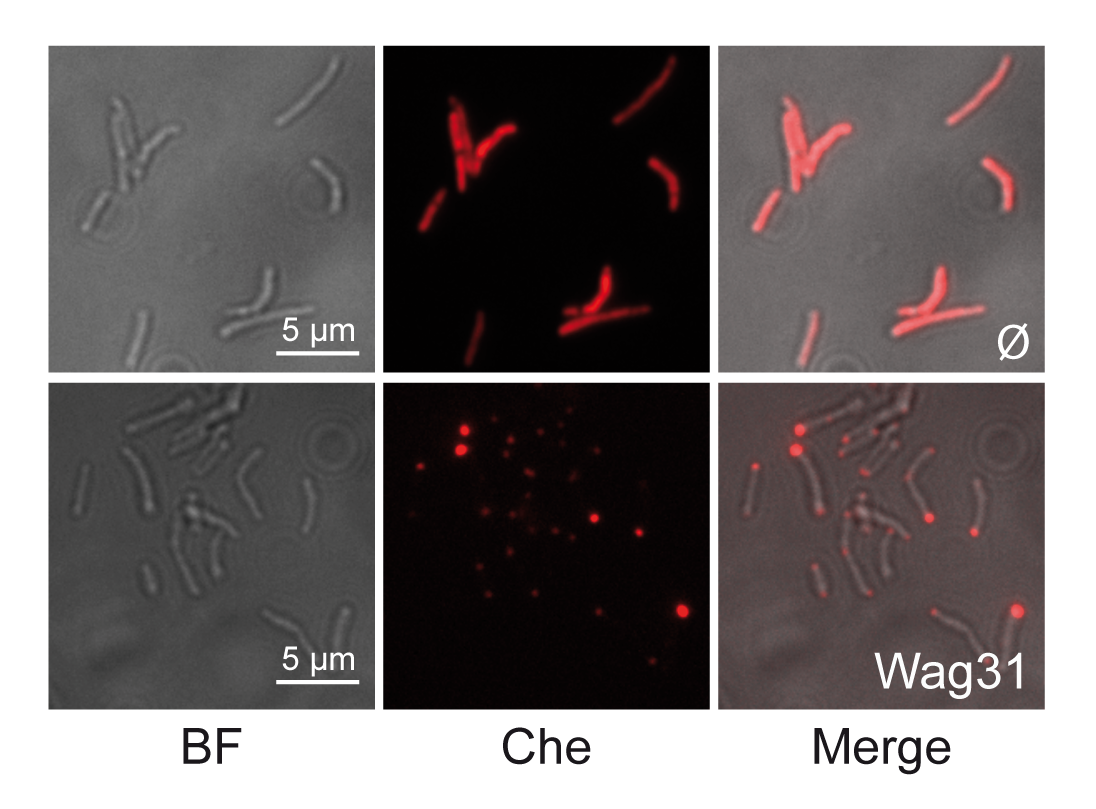

Supplement: Figure S3 — Localization of mCherry-Wag31 in Msm::mcherry-wag31 . Enlarged images of wide-field microscopy experiments on mCherry-Wag31 fusion-expressing bacteria as presented in Figure 2 and Figure 4. Brightfield images (BF; leftmost column) together with mCherry fluorescence images (Che, middle column) of Msm mc2 155::pMV361::mCherry (Ø) and Msm mc2155::pMV361::mCherry-wag31 (Wag31) were acquired 6 hours after dilution of the main culture and in the presence of anhydro-tetracyclin (50 ng/mL) for the latter. The merged images (right columns) allowed visualizing the localization of Che-Wag31 mainly at one pole of the bacteria (the old pole). Total optical magnification: 630 X. Scale bar: 5 µm. (TIF) [file pone.0097148.s003.tif]

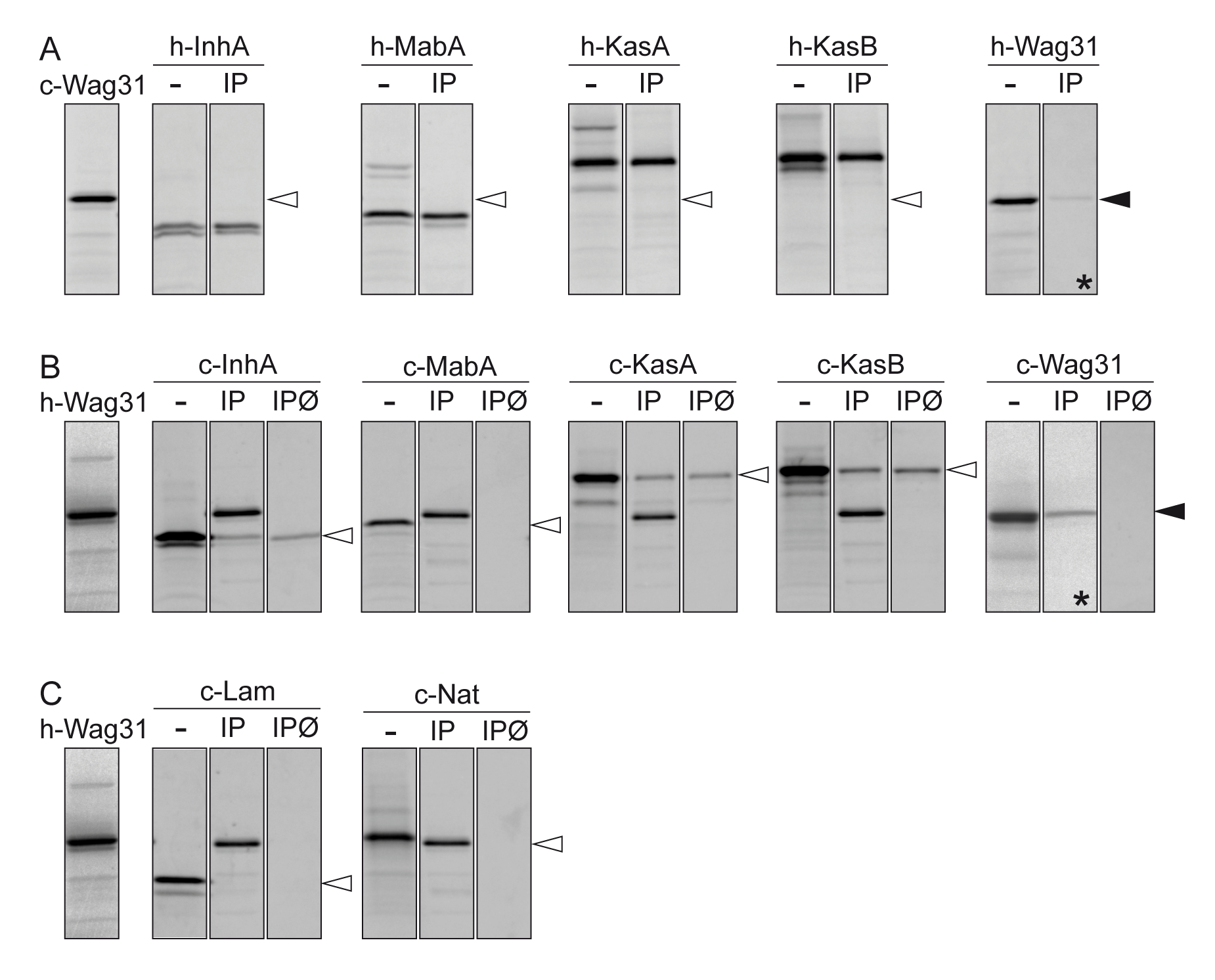

Supplement: Figure S4 — Analysis of FAS-II/Wag31 interactions by co-immunoprecipitation. L-[35S]-methionine-labeled h-proteins (HA tagged; h-protein) and c-proteins (c-Myc tagged; c-protein) from in vitro transcription translation reactions as well as the Co-IP reaction products were fractionated by SDS-PAGE (4–20%) followed by phosphorimaging analysis. The gels containing the h-Wag31 protein alone or the c-Wag31 alone are represented in the leftmost lanes. The names of the c-proteins or h-proteins used in the Co-IP experiments are indicated above each panel. These proteins were run alone (-), or after Co-IP (IP). (A) c-Wag31 was tested against h-FAS-II. (B) h-Wag31 was tested against c-FAS-II proteins; IPØ refers to a Co-IP performed between a given c-protein and a void extract (obtained with the pGAD-T7 empty vector). (C) h-Wag31 was tested against human laminC (c-Lam) with Mtb Arylamine N-acetyltransferase (c-Nat) used as negative control. The black arrowheads represent a Co-IP band while the open arrowheads indicate the position of a missing Co-IP band corresponding to a negative interaction. When the sizes of both proteins were too close to each other, non-labeled h-proteins were used for Co-IP experiments as indicated with an asterisk. (TIF) [file pone.0097148.s004.tif]

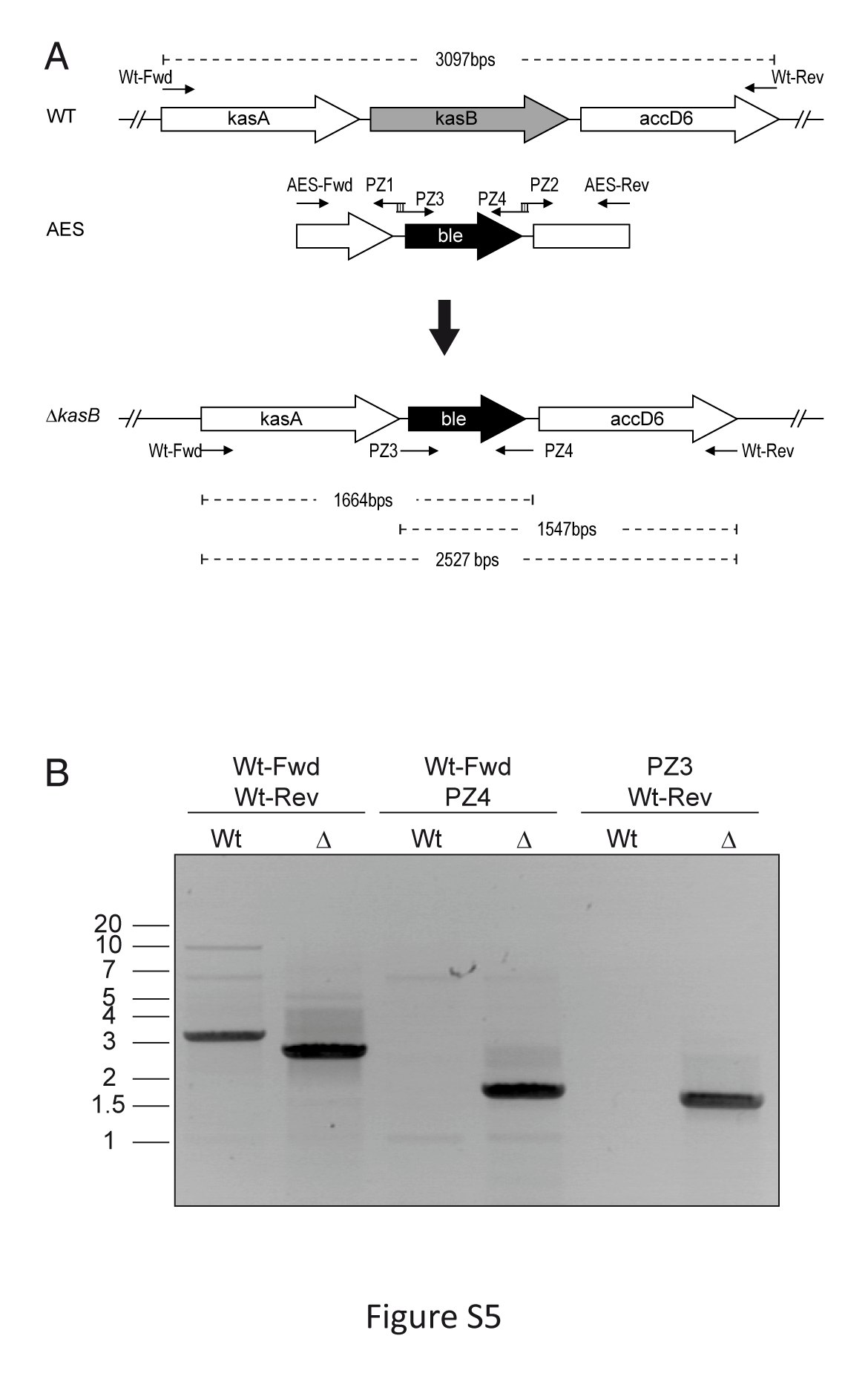

Supplement: Figure S5 — Construction and characterization of M.smegmatis Δ kasB . (A) The wild type region (WT) of the Msm chromosome comprising kasA (open arrow), kasB (grey arrow) and accD6 (open arrow) is depicted as a solid line together with the positions for hybridization of PCR primers (Wt-Fwd and Wt-Rev). The AES product synthesized by fusion-PCR is also presented with the primers used for its synthesis. The Zeocin resistance gene (Sh ble; ble) is represented as a black arrow. The structure of the chromosomal region after the allelic exchange is illustrated together with the positions of PCR primers used for its characterization. The lengths of each PCR product are indicated in base pairs (bps) and represented by dashed lines. (B) Ethydium bromide staining of an agarose gel of PCR products of wild-type (Wt) and ΔkasB (Δ) Msm genomic DNA. The primer pairs used for amplification are indicated above the gel. The migration positions of control linear DNA are indicated along with their respective sizes (in kbp). (TIF) [file pone.0097148.s005.tif]

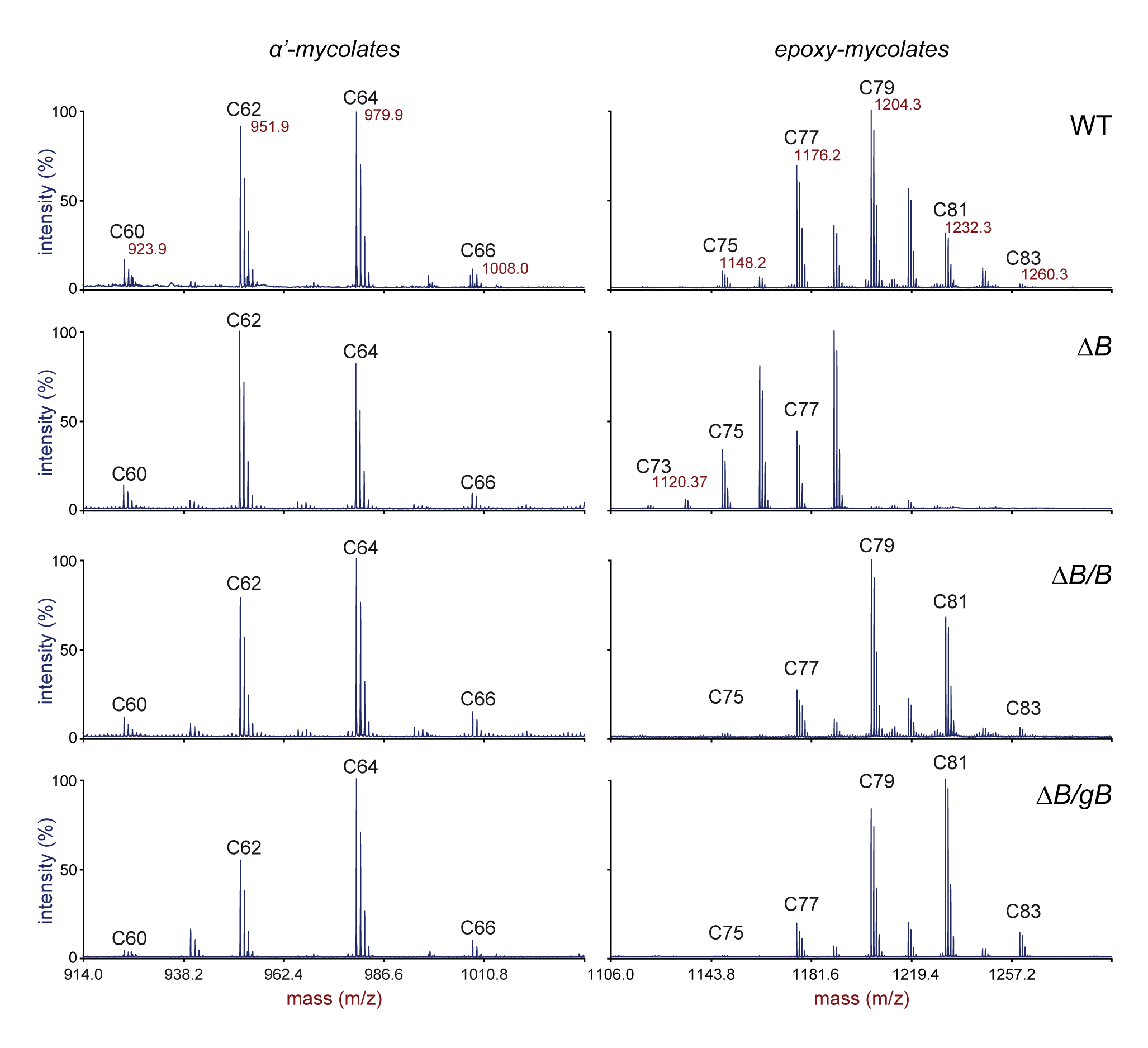

Supplement: Figure S6 — MALDI-TOF analysis of α'- and epoxy- mycolic acid methyl esters. Mass spectra of α'-mycolic acid (left column) and epoxy-mycolic acid methyl esters (right column) extracted from wild type (Msm mc2155, WT), mutant (Msm mc2155 ΔkasB, ΔB) and complemented (Msm mc2155 ΔkasB/kasB, ΔB/B; Msm mc2155 ΔkasB/gfp-kasB, ΔB/gB) strains. The accurate masses (m/z), together with the length of the even carbon-numbered epoxy-mycolates and the odd carbon-numbered α'-mycolates are indicated. (TIF) [file pone.0097148.s006.tif]
