# Supplementary material for: Mycobacterium tuberculosis Proteins Involved in Mycolic Acid Synthesis and Transport Localize Dynamically to the Old Growing Pole and Septum
Source: PLoS One. 2014 May 9;9(5):e97148. doi: 10.1371/journal.pone.0097148 (PMC4016276; doi:10.1371/journal.pone.0097148)
Supplement: Table S1 — Description of plasmids. (DOCX) [file pone.0097148.s007.docx]

| **TABLE S1. Description of plasmids** | | | |
| --- | --- | --- | --- |
| Plasmid name | Gene cloned | Vector | Origin |
| pGAD-T7::*kasA* | *kasA* | pGAD-T7 | [[16](#_ENREF_16)] |
| pGAD-T7::*kasB* | *kasB* | pGAD-T7 | [[16](#_ENREF_16)] |
| pGAD-T7::*mabA* | *mabA* | pGAD-T7 | [[16](#_ENREF_16)] |
| pGAD-T7::*inhA* | *inhA* | pGAD-T7 | [[16](#_ENREF_16)] |
| pGAD-T7::*fabH* | *mtfabH* | pGAD-T7 | [[16](#_ENREF_16)] |
| pGAD-T7::*wag31* | *wag31* | pGAD-T7 | This study |
| pGAD-T7::*nat* | *nat* | pGAD-T7 | This study |
| pGBK-T7::*kasA* | *kasA* | pGBK-T7 | [[16](#_ENREF_16)] |
| pGBK-T7::*kasB* | *kasB* | pGBK-T7 | [[16](#_ENREF_16)] |
| pGBK-T7::*mabA* | *mabA* | pGBK-T7 | [[16](#_ENREF_16)] |
| pGBK-T7::*inhA* | *inhA* | pGBK-T7 | [[16](#_ENREF_16)] |
| pGBK-T7:*fabH* | *fabH* | pGBK-T7 | [[16](#_ENREF_16)] |
| pGBK-T7::*lam* | *lamin* | pGBK-T7 | Clontech |
| pGBK-T7::*wag31* | *wag31* | pGBK-T7 | This study |
| pGBK-T7::*nat* | *nat* | pGBK-T7 | This study |
| pLAM12::*mabA* | *mabA* | pLAM12 | This study |
| pLAM12::*inhA* | *inhA* | pLAM12 | This study |
| pLAM12::*kasA* | *kasA* | pLAM12 | This study |
| pLAM12::*kasB* | *kasB* | pLAM12 | This study |
| pLAM12::*gfp* | *egfp* | pLAM12 | This study |
| pLAM12::*gfp-mabA* | *egfp* | pLAM12::*mabA* | This study |
| pLAM12::*gfp-inhA* | *egfp* | pLAM12::*inhA* | This study |
| pLAM12::*gfp-kasA* | *egfp* | pLAM12::*kasA* | This study |
| pLAM12::*gfp-kasB* | *egfp* | pLAM12::*kasB* | This study |
| pLAM12::*mmpl3-gfp* | *mmpl3* | pLAM12::*gfp* | This study |
| pLAM12*::mp3-N10-gfp* | *mmpl3-N10* | pLAM12::*gfp* | This study |
| pMV261::*wag31* | *wag31* | pMV261-Nde | This study |
| pMV361::*che-wag31* | *wag31* | pMV261::*che-wag31* | This study |
| pMV361::*che* | *mcherry* | pMV361 | This study |
