# Supplementary material for: Mycobacterium tuberculosis Proteins Involved in Mycolic Acid Synthesis and Transport Localize Dynamically to the Old Growing Pole and Septum
Source: PLoS One. 2014 May 9;9(5):e97148. doi: 10.1371/journal.pone.0097148 (PMC4016276; doi:10.1371/journal.pone.0097148)
Supplement: Table S2 — Oligonucleotide sequences of PCR primers. (DOCX) [file pone.0097148.s008.docx]

| **TABLE S2. Oligonucleotide sequences of PCR primers** | | | |
| --- | --- | --- | --- |
| Name^a^ | Sequence (5’ to 3’)^b^ | Site | Target |
| mCherry (F1) | GCAGCCATATGGTGAGCAAGGGC | *Nde*I | *mcherry* |
| mCherry (R1) | GGTGTAAGCGGCATATGCTTGTACAGC | *Nde*I | *mcherry* |
| mCherry (R2) | CGTTGGGATCCTTACTTGTACAGCTCGTCCATGCCGC | *Bam*HI | *mcherry* |
| GFP (F1) | GTGCCGCGCGGCAGCCATATGGTGAGCAAG | *Nde*I | *egfp* |
| GFP (R1) | GCCATGGCCATATGCTTGTACAGCTCGTCC | *Nde*I | *egfp* |
| GFP (R2) | AGAGTCGCGGAAGCTTTACTTGTA | *Hind*III | *egfp* |
| Wag31 (F) | CTCGAGGGGACAACATATGCCGCTTACACC | *Nde*I | *wag31* |
| Wag31 (R) | CAACCTACCAGGATCCGGCTGCCGACCTCG | *Bam*HI | *wag31* |
| Nat (F) | TCAGAATGGCCATATGGCACTGGATCTGACCG | *Nde*I | *nat* |
| Nat (R) | GGTTCGTTTGTTCGGATCCCGTTTGCCGGGC | *Bam*HI | *nat* |
| MmpL3 (F) | CAGTAAGGAGCTCATATGTTCGCCTGGTGG | *Nde*I | *mmpL3* |
| MmpL3 (R) | CACCTCACCATATGTTAAAGGCGTCCTTCG | *Nde*I | *mmpL3* |
| Mp3-N10 (F) | CAGTAAGGAGCTCATATGTTCGCCTGGTGG | *Nde*I | *mp3-N10* |
| Mp3-N10 (R) | CGCGAGAGCGCCATATGTCGACCCAGGAGG | *Nde*I | *mp3-N10* |
| Wt-Fwd | CCTGAGGTCGATCCGGACCG | - | *kasA* |
| Wt-Rev | CCACTTGGCCTGGAACTCCTCG | - | *accd6* |
| AES-Fwd | CGCGCCGGTGTCATC | - | *kasA* |
| AES-Rev | CACATCGTAGGCGCGAC | - | *accD6* |
| PZ1 | CAGTCGATCCACGTGGAGATTTACGCGATTCTTTCCTTTACC | - | *kasA* |
| PZ2 | CCACTGAGCGTCAGACCCACGTGCTCTACTCAACCGAGTTGAATGTTTC | - | *accD6* |
| PZ3 | CTCCACGTGGATCGACTGCCAGGC | *Pml*I | *sh ble* |
| PZ4 | GAGCACGTGGGTCTGACGCTCAGTGG | *Pml*I | *sh ble* |

^a^ The primer orientation, with respect to the orientation of the gene transcription, is indicated under brackets as F (forward) or R (reverse).

^b^ Restriction sites created are underlined.
